# Merging enzymatic and synthetic chemistry with computational synthesis planning

**DOI:** 10.1038/s41467-022-35422-y

**Published:** 2022-12-14

**Authors:** Itai Levin, Mengjie Liu, Christopher A. Voigt, Connor W. Coley

**Affiliations:** 1 Synthetic Biology Center, Department of Biological Engineering, Massachusetts Institute of Technology, Cambridge, MA USA; 2 Department of Chemical Engineering, Massachusetts Institute of Technology, Cambridge, MA USA; 3 Department of Electrical Engineering and Computer Science, Massachusetts Institute of Technology, Cambridge, MA USA

**Keywords:** Biosynthesis, Cheminformatics, Chemical synthesis

## Abstract

Synthesis planning programs trained on chemical reaction data can design efficient routes to new molecules of interest, but are limited in their ability to leverage rare chemical transformations. This challenge is acute for enzymatic reactions, which are valuable due to their selectivity and sustainability but are few in number. We report a retrosynthetic search algorithm using two neural network models for retrosynthesis–one covering 7984 enzymatic transformations and one 163,723 synthetic transformations–that balances the exploration of enzymatic and synthetic reactions to identify hybrid synthesis plans. This approach extends the space of retrosynthetic moves by thousands of uniquely enzymatic one-step transformations, discovers routes to molecules for which synthetic or enzymatic searches find none, and designs shorter routes for others. Application to (-)-Δ^9^ tetrahydrocannabinol (THC) (dronabinol) and R,R-formoterol (arformoterol) illustrates how our strategy facilitates the replacement of metal catalysis, high step counts, or costly enantiomeric resolution with more elegant hybrid proposals.

## Abstract (web summary)

The identification of synthetic routes combining enzymatic and non-enzymatic reactions has been challenging and requiring expert knowledge. Here, the authors describe a computational retrosynthetic approach relying on neural network models for planning synthetic routes using both strategies.

## Introduction

Enzymatic and non-enzymatic synthetic organic (“synthetic”) reactions can be synergistically combined to leverage the unique strengths of each. Many efficient hybrid syntheses have been reported from the discovery to the process scale^1–8^. Enzymes can be used to introduce stereochemistry at key points of an otherwise synthetic process. An industrially-relevant example is the evolution and use of a transaminase to selectively catalyze the formation of the chiral amine in sitagliptin, an anti-diabetes drug, from the chemically-derived pro-sitagliptin^9^. Enzymes can also catalyze reactions with superior regioselectivity. This was leveraged for the synthesis of the antiviral compound islatravir, where a purine nucleoside phosphorylase and a phosphopentomutase were evolved to catalyze the regio- and stereoselective installation of an unnatural purine moiety on a chemically^10^ or enzymatically^11^ synthesized unprotected, unnatural deoxyribose analog. Combining enzymatic and synthetic steps can unlock a more efficient process overall than using only one or the other. A striking example of this is the implementation of an ex vivo enzymatic cascade to convert chemically fixed carbon dioxide into starch at higher rates than maize^12^. In addition to enabling unique or uniquely selective transformations, enzymes can improve the environmental sustainability of a chemical process as they are renewable and biodegradable catalysts that can operate under mild conditions^13^.

However, identifying synthetic routes that use both enzymatic and synthetic organic reaction steps remains a largely manual, intuition-driven process despite the emergence of computer-aided synthesis planning (CASP) tools.^14–17^ Retrosynthesis is a search through the space of possible chemical precursors. The starting position is a target molecule and the goal is to find a path to viable starting materials. The search space grows exponentially with the search depth, so a brute force enumeration of all precursors quickly becomes computationally intractable. Most CASP algorithms emulate chemists’ retrosynthetic analysis process; starting from the target molecule, the algorithms recursively generate the most plausible precursors until pathways to suitably simple starting materials are found. As has been previously reviewed^18–23^, CASP tools differ along many axes, including how one-step retrosynthetic moves are proposed, the class of models used to rank single-step retrosynthetic suggestions, what is considered an acceptable starting material, and the algorithms used to efficiently piece together single steps to navigate a retrosynthetic search space.

A primary differentiator is whether CASP methods are template-based or template-free. Template-based models assign scores to a pre-defined set of reaction templates—generalized reaction rules—which can be applied to the input target molecule to produce precursors. When templates are algorithmically extracted, each reaction suggested by a template-based model can be linked to precedent examples to be interpreted and reviewed by a chemist. Examples of template-based synthetic organic tools include ASKCOS,^24^ which uses a set of templates automatically extracted from Reaxys, and Synthia^21^, which uses expert-curated templates; analogous data-driven and expert bioretrosynthetic tools include Retropath^25,26^, which uses templates extracted from metabolic pathway databases^27^ and RetroBioCat^28^, which focuses on industrially-relevant biocatalytic reactions. Template-free models instead learn to generate reactant molecules from an input product molecule end-to-end^29,30^. The lack of pre-defined reaction rules theoretically allows template-free models to predict novel reactions, but complicates the task of linking predictions to existing reaction data. Examples of this approach include IBM’s RXN^31,32^ and BioNavi-NP^33^, both of which employ sequence-to-sequence Transformer models^32^ to generate precursor SMILES strings directly from product SMILES strings.

Data-driven CASP tools are limited in finding hybrid synthesis routes because distinct sets of CASP software tools have been designed for fully synthetic organic synthesis planning^24,32,34–38^ and for fully enzymatic synthesis planning^16,26,33,39^. CASP tools such as ASKCOS^24^ and AiZynthFinder^37^ were developed based on sets of reactions such as the USPTO^40^ or Reaxys^41^ where enzymatic reactions represent a fraction of the total dataset (e.g., Reaxys contains ~5 × 10^4^ enzymatic reactions compared to >10^7^ total reactions), whereas enzymatic CASP tools such as BNICE.ch^42^, RetroPath^25,26^, or the similarity-based retrosynthesis tool from ref. 43 use reaction databases such as the Kyoto Encyclopedia of Genes and Genomes (KEGG)^44^, MetaNetX^45^, or Rhea^46^ that contain exclusively enzymatic reactions. Stitching together the results from a synthetic and enzymatic CASP search for the same molecule is insufficient, as part of the challenge of hybrid synthesis planning is identifying routes where one set of reactions leads to an intermediate that can be used by the other set, yielding a route that would have remained undiscovered if only one set of reactions were considered at a time. Probst et al.^31^ use transfer learning to pretrain on synthetic reactions and fine-tune enzymatic reactions, such that the model used can suggest non-enzymatic reactions if it predicts low confidence for enzymatic suggestions. RetroBioCat allows users to manually generate hybrid networks. However, no tool is designed to automatically search hybrid retrosynthesis networks. New CASP algorithms are needed to integrate and balance the two complementary synthesis strategies.

**Fig. 1:**
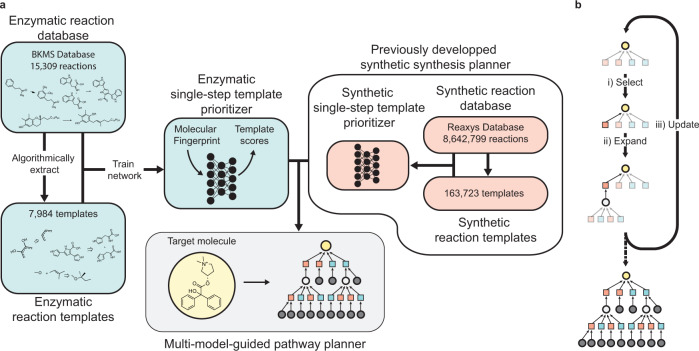
Machine learning approach to hybrid synthesis planning. **a** Development workflow of the hybrid synthesis planner. A database of enzymatic reactions was parsed into machine-readable format. Reaction templates were algorithmically extracted from the reactions in the database. A neural network template prioritizer^47^ was trained to predict the reaction template associated with each product molecule in the reaction database. The enzymatic template prioritizer and a previously trained synthetic template prioritizer^24^ are used in tandem to predict hybrid synthesis plans. **b** Multi-model-guided tree search strategy used to explore the retrosynthetic search space for multi-step synthesis planning from an input molecule (yellow circle). The possible retrosynthetic reaction templates (squares) are scored using template prioritizer neural networks. Different colors correspond to different template sets (e.g., synthetic and enzymatic). (i) The leaf node from the highest-scoring path is selected. (ii) The selected retrosynthetic template is applied to the product molecule to generate the predicted precursor. The precursor is added to the search tree and the retrosynthetic templates are scored by their corresponding template prioritizer with the precursor as input. (iii) Visit counts are updated for the explored nodes. The visit counts are used in scoring to balance exploration and exploitation. Steps i, ii, and iii are repeated until a stopping criterion for the search is met. All pathways that connect the input molecule to allowed starting materials (gray circles) are returned.

Here, we introduce a synthesis planning algorithm to generate multi-step synthesis plans that leverage the breadth of known synthetic and enzymatic chemistry (Fig. 1). We trained a template-based, enzymatic retrosynthesis neural network^47^ using enzymatic reaction data from the BKMS database^48^ to rank single enzymatic retrosynthetic steps. We show that the chemistry captured by this model expands upon chemistry captured by the synthetic chemistry retrosynthesis model from ASKCOS^24^, adding 4169 unique templates. We then designed a multi-step search algorithm that uses both the enzymatic retrosynthesis model and a synthetic retrosynthesis model to prioritize the possible retrosynthetic steps in a way that balances the exploration of enzymatic and synthetic steps. We find that this hybrid search identifies routes to molecules for which no routes are found using only enzymatic or synthetic organic chemistry. Further, the hybrid search identifies shorter pathways where enzymatic steps replace multiple synthetic steps. Finally, we demonstrate how our search algorithm can suggest promising hybrid synthesis plans which were not found otherwise, using dronabinol and arformoterol as case studies.

## Results

### Single-step enzymatic retrosynthetic expansion

An enzymatic reaction dataset was curated from the BKMS^48^ database. BKMS contains approximately 37,000 enzyme-catalyzed reactions aggregated from BRENDA^49^, the Kyoto Encyclopedia of Genes and Genomes (KEGG)^44^, Metacyc^50^, and SABIO-RK^51^. We processed the reaction data by removing biological cofactors, converting the reactions to standardized SMILES^52^ strings, and performing atom-atom mapping to track which atoms in the reactants corresponded to which atoms in the products of each reaction (see Methods). Our final dataset contains 15,309 unique, single-product, atom-mapped reaction SMILES strings.

**Fig. 2:**
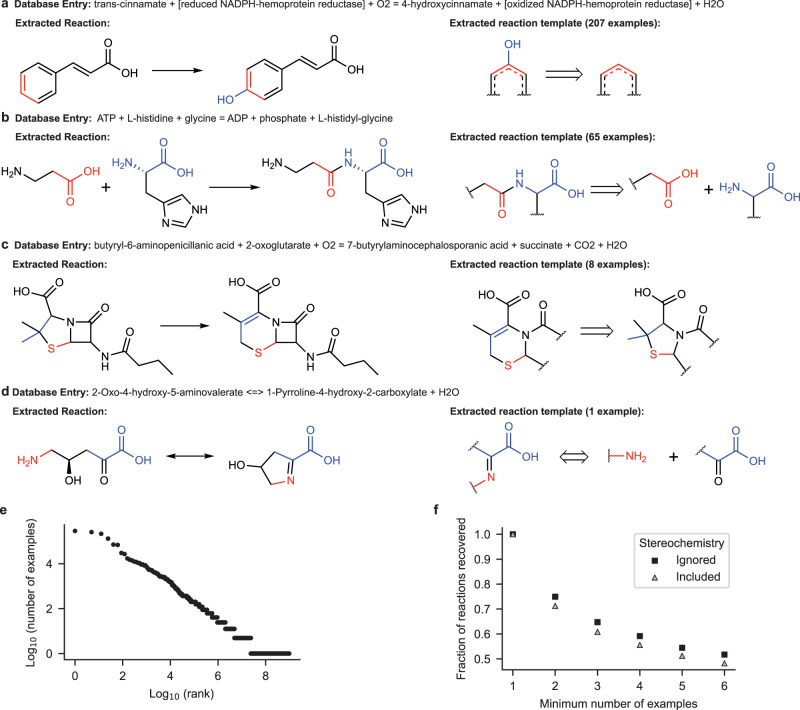
Reaction templates automatically extracted from the BKMS biochemical reaction database. **a**–**d** Examples of reactions and reaction templates extracted from the BKMS database. Cofactor reactant-product pairs (e.g., SAM and SAH) were automatically identified and removed. Chemical names were converted to SMILES strings, shown here by molecular structures. Reaction templates were automatically extracted from the atom-mapped SMILES string as SMARTS strings, shown here as retrosynthetic reaction fragments. Reaction rules are linked to the reaction database, so metadata such as associated enzymes and EC number for the reaction can be easily retrieved. **e** The number of examples for a reaction template in the reaction database as a function of the rank of the template’s popularity. **f** Fraction of the reactions in the BKMS database that can be described by reaction templates that have greater than a threshold number of examples.

Using RDChiral^53^, reaction templates summarizing the chemistry of each reaction were automatically extracted from these atom-mapped reactions as generalized SMARTS strings (examples shown in Fig. 2a–d). A total of 7984 unique reaction templates were sufficient to describe the 15,309 enzymatic reactions. This method generates a single template per reaction with chiral information and a heuristically determined, variable amount of context around the reaction center as opposed to RetroRules^27^, which stores multiple fixed-diameter templates per reaction. The generalized reaction templates approximate the range of possible chemical transformations that enzymes can catalyze by representing only the reaction center and its adjacent context from the reactant and product. This template-based approach was chosen to maintain a link between retrosynthetic suggestions and precedent reactions from the database; this link makes the model’s suggestions more interpretable and actionable as starting points for enzyme selection and optimization.

When building template-based models, a common post-processing step is to remove templates for which too few precedent examples exist. In our enzymatic reaction dataset, nearly 80% of reaction templates have only one precedent (Fig. 2e). Even if rare reactions were assigned more generalized templates and stereochemical information was removed as described in the methods section, requiring that extracted templates have *n* > 1 precedent would make the filtered set of templates unable to describe nearly 20% of reactions in the database. (Fig. 2f). Hence, we did not remove templates based on the number of precedents to maximize the diversity of enzymatic reactions that were captured.

For each enzymatic reaction, the product molecule and the extracted reaction template used in the dataset to synthesize that molecule were used as input-label pairs to train a multi-layer perceptron (MLP) classification model^47^. The MLP template prioritizer model was trained to predict which template was used to synthesize each product in the training set based on the product’s molecular structure. At inference, given a new molecular structure, the template prioritizer outputs a softmax normalized score for each of the 7984 reaction templates that can be interpreted as the probability that a given template is the best retrosynthetic move from the target molecule. After tuning the model’s hyperparameters using distinct training and validation sets, an MLP model was trained on all of the available data with a fixed number of epochs. This model was used for all subsequent analyses using synthetic targets selected from ZINC, MOSES, and FDA-approved drugs. Including all of the available data from the BKMS dataset improved the model’s ability to predict rare reactions during multi-step pathway prediction.

### Comparing enzymatic and synthetic transformations

A simple comparison between the size of the chemical reaction template set (163,723) and the enzymatic reaction template set (7984) supports the observation that synthetic organic chemistry enables a much broader set of transformations than known enzymatic chemistry. However, it is not obvious whether enzymes catalyze different reactions or simply catalyze reactions with improved specificity and efficiency. We sought to better understand what fraction of the reactions in the BKMS dataset are captured by the reaction templates in the Reaxys dataset and whether including enzymatic reactions in a retrosynthetic search could potentially expand the accessible chemical space.

To identify which reactions from BKMS comprise “unique” chemistry, we assessed whether any synthetic reaction templates from Reaxys could reproduce the same reactants given the product molecule. To this end, all of the synthetic reaction templates were applied to each of the product molecules from our BKMS dataset. If any of the reaction templates reproduced the original reactant molecule(s), the enzymatic reaction was marked as recovered and, therefore, not unique. Of the 14,601 single-step, non-spontaneous, non-generic reactions in the enzymatic reaction database, 9095 were recovered with synthetic reaction templates (not considering charge or stereochemistry). Enzyme catalysts may offer enhanced selectivity for these processes, but the chemical transformation could be achieved  without enzymes.

**Fig. 3:**
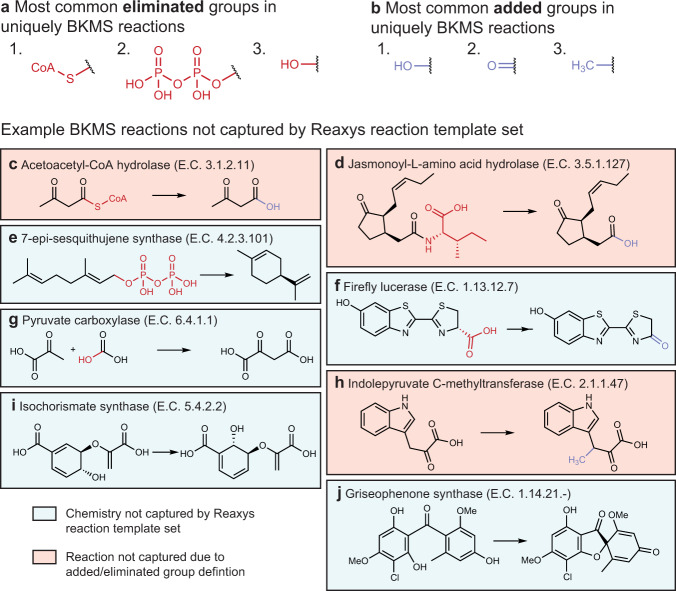
Comparison of synthetic and enzymatic reaction sets. Most commonly, **a** eliminated and **b** added substructures in reactions from the BKMS dataset that were not captured by the Reaxys dataset reaction templates. **c**–**j** Reactions from the BKMS dataset that were not recovered by applying any of the Reaxys dataset reaction templates to the product molecules. Reactions where the underlying chemistry exists in the Reaxys dataset but the reaction is not captured because of how eliminated/added groups are handled are highlighted in orange and reactions which are truly not captured by the chemistry in the Reaxys dataset are highlighted in blue.

Of the remaining 5506 enzymatic reactions (corresponding to 4169 unique reaction templates), it is likely that some could be achieved with synthetic organic chemistry. Certain reagents, cofactors, and leaving groups are omitted from reaction definitions so reactions can be modeled as single-product (Methods). This is required when performing iterative retrosynthesis. Thus, certain atoms appear only in the reactant side or product side of a reaction definition (Fig. 3a, b). For example, in 228 of the reactions in our BKMS dataset, the molecular structure corresponding to Coenzyme A appears in the reactants side and not the products side of the reaction. Only a single one of these reactions was recovered by the chemical reaction rules (Supplementary Fig. 1). This is not because the hydrolysis of a thioester bond represents uniquely enzymatic chemistry. Rather, in the Reaxys dataset, in the reaction rules representing the hydrolysis of thioesters, the acyl group is treated as the eliminated group and the product that is kept is the thiol, whereas in this work, because of our automated identification of common biological cofactors, the thiol (specifically CoA) is defined as the eliminated group and the acyl group is kept (Fig. 3c). This is more consistent with the role of CoA in biochemical reactions. The presence of eliminated and added groups confounds the automated comparison of chemical transformations across datasets, as it makes truly unique chemistry indistinguishable from the consequences of choices made during the dataset preparation (Fig. 3c–h).

To estimate a lower bound on the number of unique enzymatic transformations, we identified that of the 3466 enzymatic reactions with no addition or loss of heavy atoms between reactants and products (e.g., excluding reactions with leaving groups), 968 reactions could not be described by the Reaxys reaction templates, corresponding to 824 unique enzymatic reaction templates. Partially due to the constraints placed on this set of reactions, these are predominantly unimolecular templates that include oxidation, reduction, isomerization, and intramolecular cyclization reactions. We show two examples, the reaction of the isochorismate synthase and griseophenone synthase in Fig. 3i, j. This suggests that the enzymes expand the scope of possible organic transformations in the synthetic chemist’s toolbox and do not merely offer an alternate set of conditions for existing reactions.

### Hybrid route search guided by enzymatic and synthetic models

To explore the retrosynthetic search space efficiently, we expanded on the tree search algorithm implemented in ASKCOS^24^. Whereas the original algorithm uses one template prioritizer model to score the potential retrosynthetic moves at each step and guide the search, the algorithm developed in this work can use arbitrarily many models to guide a search. A search tree rooted at the input target molecule is constructed by the iterative selection, update, and expansion (Fig. 1b) (Methods). At the expansion step, our algorithm scores the enzymatic templates and synthetic chemistry templates using their corresponding template prioritizer models. The scores are normalized with a softmax function such that the sum of all the scores for a given template set is 1. The distinct template sets are then combined and sorted based on these scores. This means that at each step of retrosynthetic expansion, moves from either template set can be selected.

**Fig. 4:**
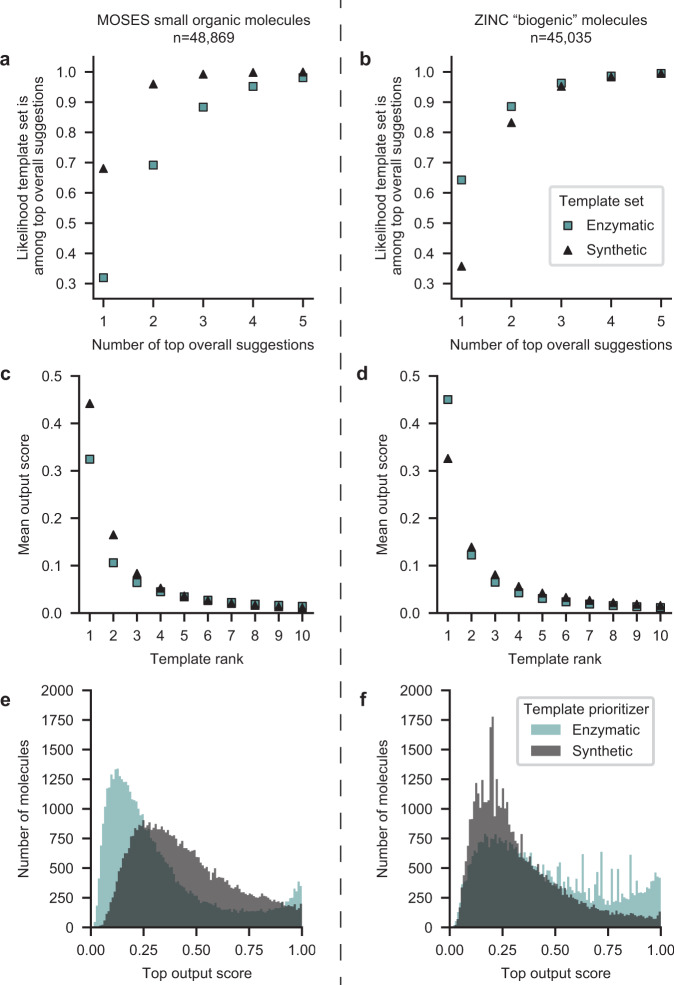
Distribution of output scores from the synthetic chemistry and enzymatic one-step retrosynthesis models. Comparison of predicted output scores (probabilities) from the synthetic chemistry and enzymatic template prioritizers illustrate their balance for different chemical spaces. The likelihood that at least one synthetic chemistry template (black) or enzymatic template (blue) will rank among the top templates when templates from both sets are combined and sorted by their probabilities for **a** small organic molecules from the MOSES dataset and for **b** biogenic molecules from ZINC. Means for the top ten probability scores using the enzymatic (blue square) and organic (black triangle) template prioritizers on **c** the MOSES subset and on **d** the ZINC subset. Distribution of top-1 probability scores using the enzymatic (blue) and organic (gray) template prioritizers when the scores are computed for **e** the MOSES subset and for the **f** ZINC subset.

The multi-model search algorithm directly compares the scores from the template prioritizer models to decide whether to explore a synthetic or enzymatic step. To identify hybrid pathways, this strategy relies on the scores (probabilities) from the two models being scaled appropriately. We show that this is the case for our models by comparing the scores of models’ top-1 recommendations for two external test sets: 48,869 small organic molecules from the MOSES dataset^54^ and 45,035 molecules annotated as biogenic (natural products) from the ZINC15 catalog^55^ that were not seen by either model during training. An enzymatic reaction template was in the top 3 suggestions for 88% of the small organic molecules and 96% of the natural product molecules. Conversely, a synthetic reaction template was in the top three suggestions for 99% of small organic molecules and 95% of natural product molecules (Fig. 4a, b). Hence, for most of the molecules in these sets, both synthetic and enzymatic steps would be considered in a pathway search that considers at least three possible moves from a molecule, allowing the algorithm to find hybrid plans when appropriate search parameters are selected.

An intuitive, slight bias appears when comparing the scores from the two models on the different chemical spaces. For small organic molecules, the top retrosynthetic proposal is more likely to be synthetic than enzymatic (Fig. 4c, e). Conversely, for the natural products, the top retrosynthetic suggestion is more likely to be enzymatic (Fig. 4d, f). This trend suggests that the models display a (justified) bias that leads to the prioritization of enzymatic chemistries for molecules that are more structurally similar to natural products.

**Fig. 5:**
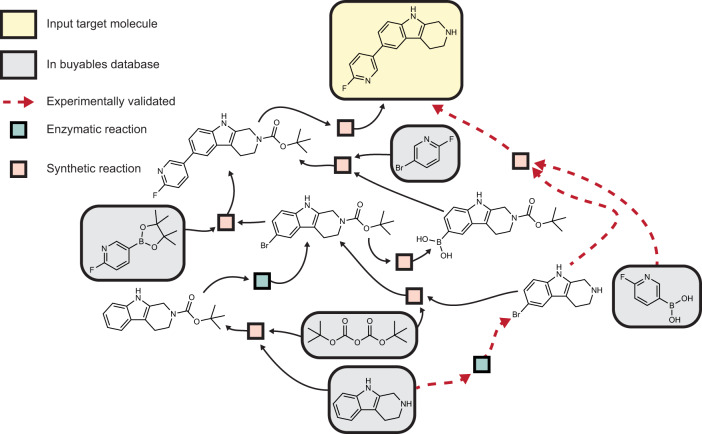
Example hybrid pathway search results for fluoropyridinyl tryptoline. Pathways displayed from a pathway search using the enzymatic and synthetic template prioritizers (see Methods and SI for more details). Some reactions are removed from the graph for visual clarity. The recovered experimentally validated pathway from ref. 56 is highlighted with red dashes.

We demonstrate how the interaction of the two models recovers the hybrid synthesis of fluoropyridinyl tryoptoline as synthesized by ref. 56 The retrosynthetic paths that reached viable starting materials are shown in Fig. 5, where the allowed starting materials were a set of buyable compounds from eMolecules and Sigma-Aldrich. The experimental pathway was recovered and is highlighted in the figure. None of the enzymatic reactions shown are present in the model’s training data, meaning that the model was able to generalize to unseen products and intermediates. The template for the enzymatic aryl bromination links back to 8 reactions from the reaction database belonging to EC classes 1.14.19.55, 1.14.19.58, and 1.97.1. The most similar reaction in the database is the bromination of tryptophan. The Brenda^49^ entry for this reaction points to the Uniprot entry for PyrH, a flavin-dependent tryptophan halogenase that shares a mechanism, reaction, and 38% sequence identity with the flavin-dependent tryptophan halogenase that ref. 56 used, RebH (Supplementary Fig. 2). This example offers a retrospective look at how our hybrid retrosynthesis algorithm could have been used as a successful starting point for route development.

**Fig. 6:**
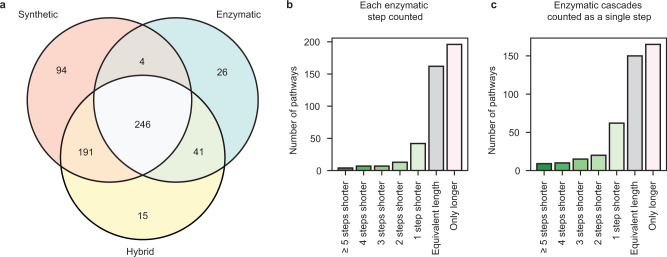
Comparison of routes found with hybrid and single-model searches. **a** Number of molecules for which synthesis routes were found out of 1000 molecules randomly sampled from the ZINC “boutique” subset. Each pathway search was performed with the same parameters using only the synthetic template prioritizer, only the enzymatic template prioritizer, or both template prioritizers at the same time (hybrid). Comparison of the number of steps in the shortest pathway found with the hybrid pathway planner (containing ≥1 enzymatic step) compared to the shortest pathway found with the organic pathway planner for molecules for which pathways were found by both (431 total) from the ZINC15 boutique sample when **b** each step in a sequence of enzymatic steps is counted and **c** consecutive enzymatic steps (cascades) are counted as a single synthetic step.

To better understand how the search space of our multi-model algorithm compared with that of the single-model algorithm, we generated a dataset of predicted synthetic routes to 1000 molecules chosen at random from the “boutique” subset of ZINC15 using a search guided by just the synthetic, just the enzymatic, or both template prioritizer models. We compared the number of molecules for which each search was able to identify synthesis pathways and the number of steps in the shortest synthesis pathway found to each target molecule (Fig. 6).

Out of the 1000 molecules from ZINC, given the same search parameters, including wall time (Supplementary Table 2), the enzymatic synthesis planner found pathways to 317, the synthetic planner found pathways to 535, and the hybrid planner found pathways to 493 (Fig. 6a). The enzymatic path planner finding paths to the smallest number of molecules is expected given that the set of enzymatic templates represents the most limited transformation space (7984 reaction templates compared to 163,723 synthetic organic reaction templates). That the hybrid planner finds routes to fewer compounds than the synthetic planner is also not wholly surprising; the hybrid path planner, in principle, can find a superset of paths found by the other two planners, but this will not be the case when performing a time-limited search. Including a larger number of templates in the search increases the number of possible moves considered in the search and can lead the search algorithm in unproductive directions. Nevertheless, when comparing the targets reached with just the synthetic search and the hybrid search, the hybrid search found routes to 56 molecules, for which none were found with the synthetic search. Of these, all of the routes found to 11 molecules would not be possible without the reaction templates in BKMS (Supplementary Fig. 3), and six molecules required transformations that were present in the constrained set of 824 uniquely enzymatic reactions. Routes to 15 molecules were found for which no routes were found with either individual strategy (Supplementary Fig. 4). This demonstrates that the hybrid search navigates a different retrosynthetic search space than the individual model searches in a manner that is beneficial for certain molecular targets.

Another metric by which to compare the outputs from the different search strategies is by the total number of reactions in the synthesis plans. Fewer reactions correlate with fewer reagents and fewer purification steps, which in turn correlates with cheaper, more efficient syntheses^57^. The length of a pathway is an incomplete measure of quality. However, the only difference between the purely synthetic and hybrid search is the inclusion of enzymatic steps, so path lengths are particularly informative when comparing pathways in this constrained context. We compare the number of reactions in the shortest pathways found with both the hybrid and the synthetic strategy (431 molecules total) (Fig. 6b); routes from the hybrid planner were required to have at least one enzymatic reaction. This analysis shows that the hybrid synthesis planner returned an improved shortest path for 73 molecules (17%) and the shortest path of equal length for 162 molecules (38%) out of the 431 boutique molecules. Because successive enzymatic reactions can often proceed in the same solvent without isolation of reaction intermediates, we perform a second analysis comparing step count when counting linear sequences of enzymatic steps as a single step (Fig. 6c). Recent experimental examples of this strategy include the ex vivo syntheses of the antiviral islatravir^11^ and monoterpene commodity chemicals^58^. Considering this perspective, the hybrid synthesis planner found improved shortest paths for 116 (27%) molecules and shortest paths of equal length to 150 (35%) out of the 431 molecules for which both synthetic and hybrid pathways were found.

### Case study: dronabinol

**Fig. 7:**
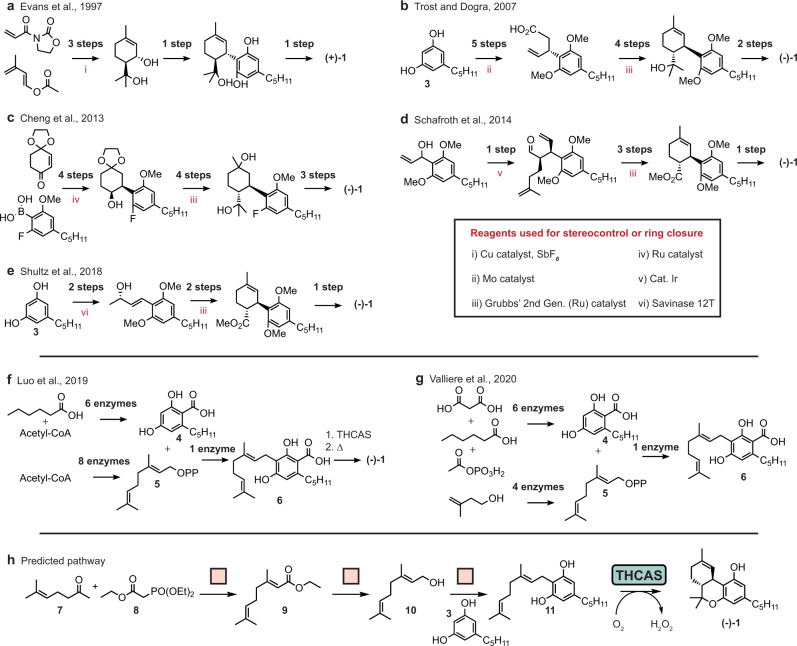
Previously published and newly proposed syntheses of dronabinol. **a**–**g** Overview of published enantioselective syntheses of dronabinol. **h** Shortest hybrid synthesis plan returned. Dronabinol does not appear in the training set of the enzymatic retrosynthesis model. THCAS tetrahydrocannabinolic acid synthase.

Dronabinol (**(−)−1**) is the generic trade name for (−)−Δ^9^ tetrahydrocannabinol (THC), indicated for the treatment of anorexia in patients with AIDS and nausea in cancer patients undergoing chemotherapy. It is the decarboxylated form of tetrahydroxycannabinolic acid (THCA), one of 113 cannabinoids naturally produced by the cannabis plant, and represents the main psychoactive agent derived from cannabis^59^. Synthetic routes to dronabinol have been established, using synthetic chemistry^60–65^, enzymatic chemistry^66,67^, and a combination of both^60^ (summary of synthesis routes in Fig. 7a–g). Briefly, the synthetic syntheses rely on transition-metal catalysts (Cu, Ir, Cr, Mo, and Ru) to set the stereocenters and to form the cyclohexene ring structure either preceding or following ligation to olivetol (**3**) (or a derivative). Alternatively, the fully enzymatic synthesis of (−)−Δ^9^ THCA was carried out in *S. cerevisiae* by ref. 66 and the enzymatic synthesis of cannabigerolic acid (CBGA, (**6**)), the immediate metabolic precursor to THCA, has been performed in vitro by ref. 67.

We performed an automated multi-step retrosynthetic analysis of dronabinol using an enzymatic, synthetic, or hybrid search. Pathways connecting dronabinol to buyable building blocks were found with the purely enzymatic and hybrid search but not with the purely synthetic search when limiting the search depth to 6. This is consistent with the fact that the published synthetic organic synthesis routes to dronabinol require more than six steps to reach buyable building blocks in our database.

The shortest and qualitatively most promising proposed synthesis route was identified with the hybrid search (Fig. 7h). The pathway first constructs geraniol (**10**) starting from cheap starting materials **7** and **8** with a Horner–Wadsworth–Emmons reaction followed by a reduction to form **10**. Next, olivetol (**4**) is alkylated with **10** to form cannabigerol **11**. This condensation can be achieved with alumina^68^. The final step is a direct enzymatic ring closure to complete the synthesis of **(−)−1**. There is a single precedent example for this template; following the recommendation back to literature precedent, the recommended enzyme is THCA synthase (EC number 1.21.3.7).

This route is promising as it suggests synthetic chemical transformations to efficiently construct **11**, which would require many enzymes to access from suitable building blocks, and suggests an enzymatic step to both close the ring and set the stereochemistry. All of the routes that were found in our retrosynthetic search relied on the reaction template extracted from the THCA synthase reaction to form the cyclohexene ring. This reaction is uniquely enzymatic and offers a “shortcut” compared to the synthetic chemistry approach with no need for transition-metal catalysts.

Although the THCA synthase reaction with **11** as the substrate is not in the training set, the model believes it may be possible to achieve this transformation enzymatically and the generalized reaction rule extracted from the THCAS reaction fits this product. It has been previously reported that the wild-type THCA synthase does not show activity on **11** and requires the carboxylated analog **6** under the same reaction conditions as the native reaction^69^. However, it remains plausible that a variant of the enzyme could catalyze the desired reaction. Given that the carboxylic acid of **6** is not believed to be directly involved in the catalytic mechanism and that the THCAS residue suspected to interact most strongly with the carboxylic acid is not strictly necessary for enzyme activity^70^, it remains possible that modifying the enzyme’s binding pocket, for example, could confer activity on the decarboxylated substrate **11**. The suggested pathway motivates an enzyme engineering effort to identify a novel variant of THCA synthase capable of catalyzing the proposed reaction on **11** to efficiently access **(−)−1**.

### Case study: arformoterol

**Fig. 8:**
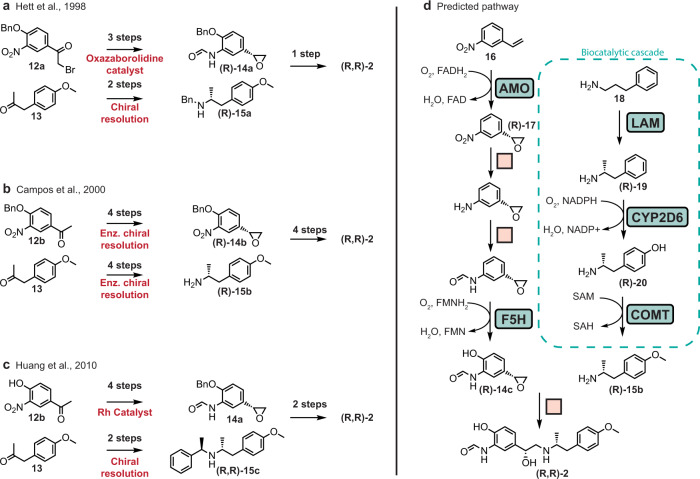
Previously published and newly proposed syntheses of arformoterol. **a**–**c** Overview of published syntheses of arformoterol. **d** Proposed hybrid synthesis plan. Enzyme names were assigned to steps based on the most structurally similar precedent reaction for reference. Arformoterol does not appear in the training set of the model. AMO alkene monooxygenase, F5H ferulate 5-hydroxylase, LAM lysine 5,6-aminomutase, CYP2D6 Cytochrome P450 2D6, COMT catechol *O*-methyltransferase.

Arformoterol (R,R-formoterol) is an enantiopure long-acting *β*_2_ adrenoreceptor agonist prescribed as a bronchodilator for patients with chronic obstructive pulmonary disease (COPD). Few routes have been reported to achieve the diastereomerically pure (R,R) form of formoterol. The three most recent reports all follow a similar, convergent logic (Fig. 8a–c)^71–73^. In one branch of the synthesis, an enantiomerically pure epoxide (**(R)-14a-b**) is synthesized from acetophenone (**12a-b**). In the other branch, an enantiomerically pure *α*-methylphenethylamine (**(R)-15a-c**)is synthesized from 4-methoxyphenylacetone (**13**). The epoxide and amine are reacted in a ring-opening reaction to form a diastereomerically pure amino alcohol which is then subjected either to deprotection reactions (refs. 71, 73) or additional functional group interconversions (ref. 72) to yield **(R,R)-2**.

As with the previous case study, the automated multi-step retrosynthetic analysis of arformoterol was run using the three different search strategies. Pathways were found by the synthetic and hybrid searches, but not the enzymatic search. A manual inspection of the 18 routes returned from the hybrid search showed that they followed a similar logic to the published routes: syntheses of a chiral amine and a chiral epoxide followed by epoxide ring-opening (or a similar nucleophilic substitution), sometimes followed by deprotection and tailoring until the formamide, hydroxyl, and methoxy groups were installed in the proper positions (Supplementary Fig. 6). Figure 8d shows one of the predicted synthesis routes that proposes an enzymatic cascade to form the chiral amine and an enzymatic epoxidation step in the formation of the chiral epoxide. Of the proposed routes, this was the most novel compared to the published routes. The introduction of enzymatic reactions unlocked building blocks that were neither found with the synthetic search nor previously reported. The suggestion of a biocatalytic cascade from **18** to **(R)-15b** is promising as it may allow the formation of the chiral amine and the installation of the methoxy group on the aryl ring of **(R)-15b** to take place in one pot with no purification of intermediates.

In contrast with previously reported methods which form the chiral amine from a ketone precursor, the route in Fig. 8 suggests a reaction rule extracted from the reactions of lysine 5,6-aminomutase (LAM, EC number 5.4.3.3) and ornithine 4,5-aminomutase (EC number 5.4.3.5) to form a chiral amine from phenylpropylamine **18**. To our knowledge, no synthetic chemical reaction has been reported to achieve the same selective cross-migration of an amine group and a hydrogen atom; as corroboration, this reaction is included in the set of unique enzymatic transformations we identified. While the usefulness of aminomutases for biotechnology has been explored in the past^74,75^, engineering adenosylcobalamin-dependent aminomutases (e.g., LAM) to act on non-natural substrates has not been extensively investigated. This suggestion highlights how the extracted reaction rules can identify motifs on products that may be accessible enzymatically but would likely require modifying a natural enzyme to accommodate the novel substrate. We acknowledge that engineering a variant of LAM for the desired reaction would not be trivial, not only because of the structural difference between phenylpropylamine (**18**) and the native substrate, lysine, but also because the native reaction produces the opposite enantiomer (S) from the desired enantiomer (R). However, the radical mechanism by which the reaction proceeds does not inherently force the production of one enantiomer over the other^76^. Previously, enzyme engineering efforts have succeeded in inverting the natural enantioselectivity of enzymes^77–80^. The addition of substrate-promiscuous, enantiocomplementary aminomutases to the biocatalysis toolset would be particularly advantageous given the popularity of chiral amines in small molecule pharmaceuticals^81^.

For the synthesis of the chiral epoxide precursor (**(R)-14c**), the search suggests leveraging an alkene monooxygenase (AMO; E.C. number 1.14.13.69) such as the enzyme from *Rhodococcus* sp. strain AD45, which has been shown to catalyze the enantioselective epoxidation of styrene and p-chlorostyrene to (R)-styrene oxide and (R)-p-chlorostyrene oxide, respectively^82^. In the full sequence from **16** to **(R)-14c**, it may be possible to perform the enzymatic epoxidation and hydroxylation steps as a cascade either before or after the chemical steps. Note that despite being able to easily identify routes with enzyme cascades after they are found, our algorithm does not explicitly promote consecutive enzymatic steps.

## Discussion

We have demonstrated a hybrid approach to retrosynthetic planning that generates promising synthesis plans with both enzymatic and synthetic steps to complex molecular targets. Deploying multiple prioritization models within a single retrosynthetic tree search was shown to balance enzymatic and synthetic reaction suggestions to discover pathways with reactions from both sets. The returned hybrid pathways can explore molecular intermediates that would not be accessible with synthetic chemistry or enzymatic chemistry alone.

By comparing the BKMS reaction dataset and the set of reaction templates previously extracted from Reaxys, we showed that the enzymatic reactions include 4196 unique transformations (of which 824 do not eliminate or add heavy atoms) that the synthetic dataset does not. While defining what it means for a transformation to be unique is not straightforward, it seems that enzyme catalysts enable chemical transformations to occur in one step that non-enzymatic reaction conditions cannot. This suggests a role for enzymes in synthetic organic chemistry not just to perform reactions with superior selectivity, but to expand the space of accessible molecules.

The diversity of enzymatic transformations also presents a challenge, as there are few reaction examples for each template; approximately 80% of templates were linked to a single reaction example in our dataset. The extent to which any retrosynthesis recommendation model is able to learn chemical trends for these rare templates is severely limited. Algorithms to generalize overly specific templates have been demonstrated to improve the performance of single- and multi-step template-based synthesis planners^83,84^. However, over-generalizing reaction templates may remove necessary chemical context and lead to fewer experimentally implementable suggestions, even if it improves model accuracy metrics. Given the complex interactions that govern enzyme-substrate compatibility, defining enzyme reaction templates at a physically meaningful level of generality may require additional information, such as reaction mechanism or binding pocket structure.

The case studies of dronabinol and arformoterol illustrate how unique enzyme chemistry can unlock routes from novel building blocks or intermediates to compounds of interest. These case studies also illustrate how the template-based retrosynthesis models may suggest enzymatic transformations that would likely require enzyme engineering or screening to implement in the lab—the sort of innovative applications of enzymes to novel substrates that expand the biocatalysis toolbox. Assessing whether an enzyme could plausibly be evolved to perform the desired reaction remains an important challenge for computational modeling and still requires expert knowledge, intuition, and experimentation. Models designed to learn the complex interactions between enzyme sequence and substrate acceptance generalize poorly even in relatively data-rich regimes^85^ and simpler molecular similarity-based methods lack the nuance needed to flag projects like the evolution of a transaminase for the synthesis of sitagliptin as worthy pursuits^43^. The nature of the algorithmically-extracted reaction templates used in this work ensures that every recommendation can be traced back to database precedents and the associated literature so users can assess the value of a suggestion based on information which may have escaped cataloging in standardized databases.

The models in this work are data-driven, so the same workflow used to balance synthetic and enzymatic synthesis steps can be applied directly to new datasets. The approach could be used to find routes with other relatively rare sets of reactions, such as “green chemistry” reactions (e.g., photochemical and electrochemical) or routes using data from multiple databases (e.g., both a public and a proprietary set of reactions). There is no theoretical guarantee that the same desirable balance between models would be observed for new models as it was between the synthetic and enzymatic models in this work, but the application of a softmax transform to the scores from each model constrains the range of the model outputs, and the empirical trend of higher model confidence for inputs that are similar to training examples seems likely to persist. Nevertheless, a balancing parameter could be introduced to manually tune the relative scores of the models if there is a known desired outcome. An additional limitation is that combining multiple models in a search expands the search space such that, given the same time limit, the hybrid search may not find pathways that a search guided by a single model would find. This further motivates exploring new techniques, such as reinforcement learning to balance model suggestions.

We believe that hybrid CASP approaches such as ours will accelerate the identification and development of new efficient synthesis routes. Enzymes can catalyze certain transformations that are not otherwise possible and increase the selectivity and efficiency of others, while synthetic chemistry offers a broader, complementary toolkit. Identifying opportunities to apply enzyme chemistry to access novel targets with our algorithm can work synergistically with experimental efforts in high throughput screening and the evolution of enzymes for the discovery of new biocatalysts.

## Methods

### Processing the BKMS database

The BKMS database^48^ is a composite database containing reactions from BRENDA^49^, the Kyoto Encylopedia of Genes and Genomes (KEGG)^44^, Metacyc^50^, and SABIO-RK^51^. The database was retrieved as a flat file with 37,235 enzymatic reactions. Reactions are represented in the form A + B + ... = C + ..., where A, B, and C are unstandardized chemical names.

Cofactor reactant-product pairs were automatically identified by computing the co-occurrence of molecule names as reactants and products (e.g., NAD+ and NADH). Product-reactant pairs that co-occur in at least ten reactions in at least 90% of the reactions they were in were removed from the reactions in which they co-occur (full list in Supplementary Table 3). This was particularly important to remove chemicals for which SMILES strings could not be found (e.g., “oxidized ferredoxin.”)

A dictionary mapping chemical names to SMILES strings was constructed using the PubChem Identifier Exchange Service^86^ and data downloaded from MetaCyc and BRENDA. This dictionary was used to obtain reaction SMILES for all reactions. Reactions were removed if SMILES were not found for all reactants and products.

Reaction SMILES were automatically atom-atom-mapped using the Reaction Decoder Tool^87^. The reverse reaction was added for all reactions that were indicated to be reversible in BKMS.

Further processing was performed to maximize the number of single-product reactions, because multi-product reactions cannot be handled in the tree search. The number of times that each molecule SMILES appeared as a product in a reaction with >1 product was counted. Iteratively, the most frequently co-appearing molecule was removed from all multi-product reactions. During this process, common side-products or leaving groups such as coenzyme A and d-glucose were removed from multi-product reactions. Finally, reactant molecules that did not have at least three mapped atoms and 15% of their mapped atoms represented in the products were removed from reactions with >1 reactant. After deduplication, the cleaned reaction database contained 18,719 cleaned, mapped reaction SMILES.

### Automatic extraction of reaction rules

Reaction templates were automatically extracted using RDChiral^53^. The template radius was set to 1 and the default special groups were included in the templates. Templates were validated by applying extracted templates back to the product using RDKit^88^ and checking that the generated reactants matched the reactants from the database. During validation, stereochemistry was ignored around atoms and bonds that were not matched by the template to avoid SMILES definition mismatches that were not relevant to the reaction. Reactions that led to invalid templates were removed. A total of 7984 valid templates were extracted from 15,309 reactions.

The template label prediction accuracy increases when there are more examples with that label in the training set (Supplementary Fig. 7). We studied how filtering out reaction templates with few examples affected the breadth of chemistry that the template set could cover. For a range of threshold values, all templates that had fewer examples than the threshold were removed from the data. Then, all templates with more examples than the threshold were applied to the products from all unlabeled reactions using RDKit with and without considering stereochemistry. If any template recovered the reactants, the reaction precedent was reassigned to that template and retained; otherwise, the reaction was removed. The relative number of reactions that were covered at different template popularity thresholds is shown in Figure 2f. Because of the substantial decrease in reactions covered when any threshold was set, templates were not filtered out based on popularity in any subsequent evaluations.

### Training the template prioritizer

The template prioritizer is an MLP model trained to score all the retrosynthetic moves for an input product molecule represented by reaction templates. The input for the template prioritizer is a 2048-bit Morgan fingerprint representation of a product molecule as implemented in RDKit^88^ with chiral features. The output is a vector of length 7984, corresponding to the number of templates. A softmax activation is applied to the final layer, such that the score across all templates sums to 1 and can be interpreted as a probability.

The data were initially split 80% in training, 10% in validation, and 10% in test sets using a previously described stratified split^89^ to ensure a more even distribution of class labels across the splits. For templates with ten or more examples, the examples were assigned at random to one of the three sets. For templates with fewer than ten examples, at random, one reaction example was assigned to the test and one to the validation set and the rest were kept in the training set. For templates with exactly two examples, one was assigned to the test set and one was assigned to the train set. For templates with a single example, the example was assigned at random to the train, validation, or test sets with a probability proportional to the size of the set.

The hyperparameters tuned were the number of hidden layers (1, 2, or 3), the size of the hidden layers (1024, 2048, or 4096), and the number of highway layers^90^ (0, 1, or 3) using a grid search. Models were trained with and without pre-training on template applicability^91^ and accuracies were calculated on the validation set. The best performance was achieved with 1 hidden layer of size 4096 and 0 highway layers. Pre-training increased the accuracy of all models tested, so the final network used in the multi-step search was pre-trained.

The single-step retrosynthesis model trained on BKMS data ranked the correct template as the top template for 19% of the product molecules from the test set and ranked the correct template in the top 10 for 53% of the test set molecules. We trained an additional model on all of the available data to maximize the diversity of template labels seen by the model. We set hyperparameters and the number of training epochs based on the tuning performed with the split dataset. This final model, trained on the entire dataset, was only used with external test sets selected independently.

### Multi-prioritizer guided tree search

The multi-prioritizer tree search uses an expanded version of the algorithm used in ASKCOS.^24^ A search tree is constructed from an input target molecule by iterative rounds of selection, expansion, and update steps. During selection, the leaf node of the highest-scoring pathway is identified by greedily traversing the tree, picking the highest-scoring children nodes until a leaf is reached. The node scores are a function of the neural network model scores MLP(**P**), the visit counts of the product node *N*_*P*_ and the child reaction node *N*_*R*_, an exploration factor *c*, and a value estimate for the reaction node *V*_*R*_. *V*_*R*_ is assigned 0 if no paths from the node reach buyable starting materials. Otherwise, it is the mean number of building blocks needed for routes that do reach buyable starting materials from the node.

1 $$\,{{\mbox{Score}}}=\frac{c\sqrt{{N}_{P}}}{1+{N}_{R}}{{\mbox{MLP}}}\,({{{{{{{\bf{P}}}}}}}})-{V}_{R}$$

The selected leaf node represents a yet-to-be-applied reaction template. During the update step, node visit counts are incremented along the path from the root node (target molecule) to the selected leaf. At the expansion step, a new precursor is generated by applying the leaf node template to its parent chemical node. With the new precursor as input, all reaction templates from each template set are scored separately by their respective template prioritizer model, then combined and re-sorted. The key difference from the original ASKCOS algorithm is that an unbounded number of template prioritizers can be used instead of exactly one. The number of suggestions grows linearly with the number of models in use. Once the expansion time is elapsed, the tree construction halts. Paths that connect the target molecule to buyable starting materials are retrieved and returned using a depth-first search.

### Buyable database

The default buyable compound database from ASKCOS was used for all pathway searches. This database contains 106,750 compounds available for less than $100/g from the vendors eMolecules and Sigma-Aldrich. An estimated price is associated with each compound.

### Benchmarking search strategies

One thousand molecules were randomly selected as targets from the set of named, “boutique” compounds from the ZINC15^55^ database. Three tree searches were performed for each molecule: one using only the enzymatic retrosynthesis model, one using the synthetic retrosynthesis model, and one using both retrosynthesis models. The parameters for all three searches were otherwise identical. The maximum search depth was ten and the maximum expansion time was 180 s (additional parameters in Supplementary Table 2). All synthesis plans were performed using Google Cloud Platform VM instances with four cores and 26 GB of memory.

### Synthesis planning

The synthesis plans for fluoropyridinyl tryptoline, dronabinol, and arformoterol were automatically generated, using the same parameters as the hybrid search performed in the benchmarking analysis. The only difference was that the search depth was capped at 4, 6, and 7 reactions, respectively for the three targets based on the expected path lengths of previously published routes. Potential enzymes were assigned to reactions from the most similar reaction precedent as defined by the Tanimoto similarity between the RDKit reaction structural fingerprints of the proposed and precedent reactions.

## Supplementary information

Supplementary Information

## Data Availability

Reaxys reaction data used to build the previously published synthetic organic model is the intellectual property of Elsevier and cannot be shared. The templates extracted from the Reaxys data and all of the BKMS data used to build the enzymatic model and associated templates is available at https://github.com/itai-levin/bkms-data^92^.

## Code availability

The source code for deploying ASKCOS is available at https://github.com/itai-levin/chemoenzymatic-askcos^93^. The code for performing the analyses in this manuscript is available at https://github.com/itai-levin/hybmind^94^. This repository contains a detailed README with instructions on how to generate the results presented in the manuscript.

## References

[1] Chakrabarty, S., Romero, E. O., Pyser, J. B., Yazarians, J. A. & Narayan, A. R. H. Chemoenzymatic total synthesis of natural products. *Acc. Chem. Res.* **54**, 1374–1384 (2021). 10.1021/acs.accounts.0c00810 PMC8210581 33600149

[2] Li J, Amatuni A, Renata H (2020). Recent advances in the chemoenzymatic synthesis of bioactive natural products. Curr. Opin. Chem. Biol..

[3] Zhang X (2020). Divergent synthesis of complex diterpenes through a hybrid oxidative approach. Science.

[4] Patel NR (2020). Synthesis of islatravir enabled by a catalytic, enantioselective alkynylation of a ketone. Org. Lett..

[5] M. Abdelraheem EM, Busch H, Hanefeld U, Tonin F (2019). Biocatalysis explained: from pharmaceutical to bulk chemical production. React. Chem. Eng..

[6] Wu, S., Snajdrova, R., Moore, J. C., Baldenius, K. & Bornscheuer, U. Biocatalysis: enzymatic synthesis for industrial applications. *Angew. Chem. Int. Ed.* **60**, 88–119 (2020). 10.1002/anie.202006648 PMC7818486 32558088

[7] Sheldon RA, Brady D, Bode ML (2020). The Hitchhiker’s guide to biocatalysis: recent advances in the use of enzymes in organic synthesis. Chem. Sci..

[8] Fryszkowska A, Devine PN (2020). Biocatalysis in drug discovery and development. Curr. Opin. Chem. Biol..

[9] Savile CK (2010). Biocatalytic asymmetric synthesis of chiral amines from ketones applied to sitagliptin manufacture. Science.

[10] Nawrat CC (2020). Nine-step stereoselective synthesis of islatravir from deoxyribose. Org. Lett..

[11] Huffman MA (2019). Design of an in vitro biocatalytic cascade for the manufacture of islatravir. Science.

[12] Cai T (2021). Cell-free chemoenzymatic starch synthesis from carbon dioxide. Science.

[13] Truppo MD (2017). Biocatalysis in the pharmaceutical industry: the need for speed. ACS Med. Chem. Lett..

[14] Struble TJ (2020). Current and future roles of artificial intelligence in medicinal chemistry synthesis. J. Med. Chem..

[15] Baum, Z. J. et al. Artificial intelligence in chemistry: current trends and future directions. *J, Chem. Inf. Model.* **61**, 3197–3212 (2021). 10.1021/acs.jcim.1c00619 34264069

[16] Hadadi N, Hatzimanikatis V (2015). Design of computational retrobiosynthesis tools for the design of de novo synthetic pathways. Curr. Opin. Chem. Biol..

[17] Lin G-M, Warden-Rothman R, Voigt CA (2019). Retrosynthetic design of metabolic pathways to chemicals not found in nature. Curr. Opin. Syst. Biol..

[18] Cook A (2012). Computer-aided synthesis design: 40 years on. WIREs Comput. Mol. Sci..

[19] Ravitz O (2013). Data-driven computer aided synthesis design. Drug Discov. Today. Technol..

[20] Johansson S (2019). AI-assisted synthesis prediction. Drug Discov. Today. Technol..

[21] Szymkuć S (2016). Computer-assisted synthetic planning: the end of the beginning. Angew. Chem. Int. Ed..

[22] Coley CW, Green WH, Jensen KF (2018). Machine learning in computer-aided synthesis planning. Acc. Chem. Res..

[23] Shen Y (2021). Automation and computer-assisted planning for chemical synthesis. Nat. Rev. Methods Prim..

[24] Coley CW (2019). A robotic platform for flow synthesis of organic compounds informed by AI planning. Science.

[25] Delépine B, Duigou T, Carbonell P, Faulon J-L (2018). RetroPath2.0: a retrosynthesis workflow for metabolic engineers. Metab. Eng..

[26] Koch M, Duigou T, Faulon J-L (2020). Reinforcement learning for bioretrosynthesis. ACS Synth. Biol..

[27] Duigou T, du Lac M, Carbonell P, Faulon J-L (2019). RetroRules: a database of reaction rules for engineering biology. Nucleic Acids Res..

[28] Finnigan W, Hepworth LJ, Flitsch SL, Turner NJ (2021). RetroBioCat as a computer-aided synthesis planning tool for biocatalytic reactions and cascades. Nat. Catal..

[29] Liu B (2017). Retrosynthetic reaction prediction using neural sequence-to-sequence models. ACS Cent. Sci..

[30] Zheng S, Rao J, Zhang Z, Xu J, Yang Y (2020). Predicting retrosynthetic reactions using self-corrected transformer neural networks. J. Chem. Inf. Model..

[31] Probst, D. et al. Biocatalysed synthesis planning using data-driven learning. *Nat. Commun.* **13**, 964 (2022) 10.1038/s41467-022-28536-w PMC8857209 35181654

[.32] Schwaller P (2020). Predicting retrosynthetic pathways using transformer-based models and a hyper-graph exploration strategy. Chem. Sci..

[33] Zheng, S. et al. Deep learning driven biosynthetic pathways navigation for natural products with BioNavi-NP. *Nat Commun.* **13**, 3342 (2022). 10.1038/s41467-022-30970-9 PMC9187661 35688826

[34] Corey E, Long A, Rubenstein S (1985). Computer-assisted analysis in organic synthesis. Science.

[35] Bøgevig A (2015). Route design in the 21st century: the IC *SYNTH* software tool as an idea generator for synthesis prediction. Org. Process Res. Dev..

[36] Segler MHS, Preuss M, Waller MP (2018). Planning chemical syntheses with deep neural networks and symbolic AI. Nature.

[37] Genheden S (2020). AiZynthFinder: a fast, robust and flexible open-source software for retrosynthetic planning. J. Cheminformatics.

[38] Mikulak-Klucznik B (2020). Computational planning of the synthesis of complex natural products. Nature.

[39] Bachmann BO (2010). Biosynthesis: is it time to go retro?. Nat. Chem. Biol..

[40] Lowe, D. Chemical reactions from US patents (1976-Sep2016). figshare 10.6084/m9.figshare.5104873.v1. (2017).

[41] Badowski T, Gajewska EP, Molga K, Grzybowski BA (2020). Synergy between expert and machine-learning approaches allows for improved retrosynthetic planning. Angew. Chem. Int. Ed..

[42] Tokic M (2018). Discovery and evaluation of biosynthetic pathways for the production of five methyl ethyl ketone precursors. ACS Synth. Biol..

[43] Sankaranarayanan K (2022). Similarity based enzymatic retrosynthesis. Chem. Sci..

[44] Kanehisa M, Goto S (2000). KEGG: Kyoto Encyclopedia of genes and genomes. Nucleic Acids Res..

[45] Moretti S, Tran V, Mehl F, Ibberson M, Pagni M (2021). MetaNetX/MNXref: unified namespace for metabolites and biochemical reactions in the context of metabolic models. Nucleic Acids Res..

[46] Bansal P (2022). Rhea, the reaction knowledgebase in 2022. Nucleic Acids Res..

[47] Segler MHS, Waller MP (2017). Neural-symbolic machine learning for retrosynthesis and reaction prediction. Chemistry.

[48] Lang M, Stelzer M, Schomburg D (2011). BKM-react, an integrated biochemical reaction database. BMC Biochem..

[49] Chang A (2021). BRENDA, the ELIXIR core data resource in 2021: new developments and updates. Nucleic Acids Res..

[50] Karp PD (2019). The BioCyc collection of microbial genomes and metabolic pathways. Brief. Bioinforma..

[51] Wittig U (2012). SABIO-RK-database for biochemical reaction kinetics. Nucleic Acids Res..

[52] Weininger D (1988). SMILES, a chemical language and information system. 1. Introduction to methodology and encoding rules. J. Chem. Inf. Model..

[53] Coley CW, Green WH, Jensen KF (2019). RDChiral: an RDKit wrapper for handling stereochemistry in retrosynthetic template extraction and application. J. Chem. Inf. Model..

[54] Polykovskiy, D. et al. Molecular sets (MOSES): A benchmarking platform for molecular generation models. *Front. Pharmacol.* **11**, 565644 (2020). 10.3389/fphar.2020.565644 PMC7775580 33390943

[55] Sterling T, Irwin JJ (2015). ZINC 15 - ligand discovery for everyone. J. Chem. Inf. Model..

[56] Durak LJ, Payne JT, Lewis JC (2016). Late-stage diversification of biologically active molecules via chemoenzymatic C-H functionalization. ACS Catal..

[57] Cornwall P, Diorazio LJ, Monks N (2018). Route design, the foundation of successful chemical development. Bioorg. Med. Chem..

[58] Korman TP, Opgenorth PH, Bowie JU (2017). A synthetic biochemistry platform for cell free production of monoterpenes from glucose. Nat. Commun..

[59] Aizpurua-Olaizola O (2016). Evolution of the cannabinoid and terpene content during the growth of *Cannabis sativa* plants from different chemotypes. J. Nat. Prod..

[60] Shultz ZP, Lawrence GA, Jacobson JM, Cruz EJ, Leahy JW (2018). Enantioselective total synthesis of cannabinoids-A route for analogue development. Org. Lett..

[61] Cheng L-J, Xie J-H, Chen Y, Wang L-X, Zhou Q-L (2013). Enantioselective total synthesis of (-)-Δ^8^-THC and (-)-Δ^9^-THC via catalytic asymmetric hydrogenation and SNAr cyclization. Org. Lett..

[62] Schafroth MA, Zuccarello G, Krautwald S, Sarlah D, Carreira EM (2014). Stereodivergent total synthesis of Δ^9^-tetrahydrocannabinols. Angew. Chem. Int. Ed..

[63] Ametovski A, Lupton DW (2019). Enantioselective total synthesis of (-)-Δ^9^-tetrahydrocannabinol via N-heterocyclic carbene catalysis. Org. Lett..

[64] Evans DA (1999). Bis(oxazoline) and bis(oxazolinyl)pyridine copper complexes as enantioselective Diels-Alder catalysts: reaction scope and synthetic applications. J. Am. Chem. Soc..

[65] Trost BM, Dogra K (2007). Synthesis of (-)-Δ^9^-*trans*-tetrahydrocannabinol: stereocontrol via Mo-catalyzed asymmetric allylic alkylation reaction. Org. Lett..

[66] Luo X (2019). Complete biosynthesis of cannabinoids and their unnatural analogues in yeast. Nature.

[67] Valliere, M. A., Korman, T. P., Arbing, M. A. & Bowie, J. U. A bio-inspired cell-free system for cannabinoid production from inexpensive inputs. *Nat. Chem. Biol.* **16**, 1427–1433 (2020). 10.1038/s41589-020-0631-9 32839605

[68] Jentsch NG, Zhang X, Magolan J (2020). Efficient synthesis of cannabigerol, grifolin, and piperogalin via alumina-promoted allylation. J. Nat. Products.

[69] Taura F, Morimoto S, Shoyama Y, Mechoulam R (1995). First direct evidence for the mechanism of Δ^1^-tetrahydrocannabinolic acid biosynthesis. J. Am. Chem. Soc..

[70] Shoyama Y (2012). Structure and function of Δ^1^-tetrahydrocannabinolic acid (THCA) synthase, the enzyme controlling the psychoactivity of Cannabis sativa. J. Mol. Biol..

[71] Hett R, Fang QK, Gao Y, Wald SA, Senanayake CH (1998). Large-scale synthesis of enantio- and diastereomerically pure (*R*, *R*)-formoterol. Org. Process Res. Dev..

[72] Campos, F., Bosch, M. P. & Guerrero, A. An effcient enantioselective synthesis of (R,R)-formoterol, a potent bronchodilator, using lipases. *Tetrahedron Asymmetry* **13**, 2705–2717 (2000).

[73] Huang L (2010). The asymmetric synthesis of (R,R)-formoterol via transfer hydrogenation with polyethylene glycol bound Rh catalyst in PEG2000 and water. Chirality.

[74] Wu B, Szymański W, Heberling MM, Feringa BL, Janssen DB (2011). Aminomutases: mechanistic diversity, biotechnological applications and future perspectives. Trends Biotechnol..

[75] Parmeggiani F, Weise NJ, Ahmed ST, Turner NJ (2018). Synthetic and therapeutic applications of ammonia-lyases and aminomutases. Chem. Rev..

[76] Maity AN, Chen Y-H, Ke S-C (2014). Large-scale domain motions and pyridoxal-5’-phosphate assisted radical catalysis in coenzyme B12-dependent aminomutases. Int. J. Mol. Sci..

[77] Kille S, Zilly FE, Acevedo JP, Reetz MT (2011). Regio- and stereoselectivity of P450-catalysed hydroxylation of steroids controlled by laboratory evolution. Nat. Chem..

[78] Zhu D (2008). Inverting the enantioselectivity of a carbonyl reductase via substrate-enzyme docking-guided point mutation. Org. Lett..

[79] Pratter SM (2013). Inversion of enantioselectivity of a mononuclear non-heme iron(II)-dependent hydroxylase by tuning the interplay of metal-center geometry and protein structure. Angew. Chem. Int. Ed..

[80] May O, Nguyen PT, Arnold FH (2000). Inverting enantioselectivity by directed evolution of hydantoinase for improved production of L-methionine. Nat. Biotechnol..

[81] Ghislieri D, Turner NJ (2014). Biocatalytic approaches to the synthesis of enantiomerically pure chiral amines. Top. Catal..

[82] van Hylckama Vlieg JET, Leemhuis H, Spelberg JHL, Janssen DB (2000). Characterization of the gene cluster involved in isoprene metabolism in Rhodococcus sp. strain AD45. J. Bacteriol..

[83] Law J (2009). Route designer: a retrosynthetic analysis tool utilizing automated retrosynthetic rule generation. J. Chem. Inf. Model..

[84] Heid E, Liu J, Aude A, Green WH (2022). Influence of template size, canonicalization, and exclusivity for retrosynthesis and reaction prediction applications. J. Chem. Inf. Model..

[85] Goldman S, Das R, Yang KK, Coley CW (2022). Machine learning modeling of family wide enzyme-substrate specificity screens. PLoS Comput. Biol..

[86] NCBI. PubChem identifier exchange service. https://pubchem.ncbi.nlm.nih.gov/idexchange/idexchange.cgi.

[87] Rahman SA (2016). Reaction decoder tool (RDT): extracting features from chemical reactions. Bioinformatics.

[88] RDKit. http://www.rdkit.org/.

[89] Fortunato, M. E., Coley, C. W. & Barnes, B. C. Machine learned prediction of reaction template applicability for data-driven retrosynthetic predictions of energetic materials. *AIP Conf. Proc*. **2272**, 070014 (2020).

[90] Srivastava, R. K., Greff, K. & Schmidhuber, J. Training very deep networks. *Advances in Neural Information Processing* (2015).

[91] Fortunato ME, Coley CW, Barnes BC, Jensen KF (2020). Data augmentation and pretraining for template-based retrosynthetic prediction in computer-aided synthesis planning. J. Chem. Inf. Model..

[92] Levin, I. bkms-data. *Zenodo*. 10.5281/zenodo.7334523 (2022).

[93] Levin, I. chemoenzymatic-askcos. *Zenodo*. 10.5281/zenodo.7334532 (2022).

[94] Levin, I. hybmind. *Zenodo*. 10.5281/zenodo.7334538 (2022).

