# Supplementary material for: Merging enzymatic and synthetic chemistry with computational synthesis planning
Source: Nat Commun. 2022 Dec 14;13:7747. doi: 10.1038/s41467-022-35422-y (PMC9750992; doi:10.1038/s41467-022-35422-y)
Supplement: Supplementary file 1 — Supplementary Information [file 41467_2022_35422_MOESM1_ESM.pdf]

Supplementary information

Merging enzymatic and synthetic chemistry with computational synthesis planning

Itai Levin, Mengjie Liu, Christopher A. Voigt, and Connor W. Coley

Supplementary Information for Manuscript titled "**Merging enzymatic and synthetic chemistry with computational synthesis planning**" by Itai Levin, Mengjie Liu, Christopher A. Voigt & Connor W. Coley

Contents

Supplementary Figure 1: Additional information from comparison of chemistry captured by the Reaxys and BKMS template sets 2

Supplementary Table 1: Precedent examples for aryl bromination reaction template 3

Supplementary Figure 2: Data for the suggested enzymatic bromination of tryptoline 4

Supplementary Table 2: Parameters used for full synthesis plan searches 5

Supplementary Table 3: Automatically determined cofactor pairs 6

Supplementary Figure 3: Molecules for which all routes found required a transformation that could not be described by the synthetic chemistry template set 9

Supplementary Figure 4: Molecules for which routes were found with a hybrid search but not with a fully synthetic or fully enzymatic search 9

Supplementary Figure 5: Screenshot of synthesis route identified for dronabinol ((-)-1) 10

Supplementary Figure 6: Screenshots for the 6 shortest hybrid synthesis routes identified for arformoterol ((R,R)-2) 11

Supplementary Figure 7: Retrosynthesis prediction accuracy on test set as a function of number of training examples seen for a template 17

Supplementary Figure 8: Accuracy metrics during training of the enzymatic one-step model 18

Supplementary Figure 9: Number of nodes explored for multi-step retrosynthetic search with different search strategies 19

Supplementary Figure 10: Comparing interactive retrosynthetic search results to experimental pathways for islatravir and sitagliptin 20

References 22

Supplementary Figure 1: Additional information from comparison of chemistry captured by the Reaxys and BKMS template sets

a Examples of reactions from BKMS that were captured by the Reaxys template set

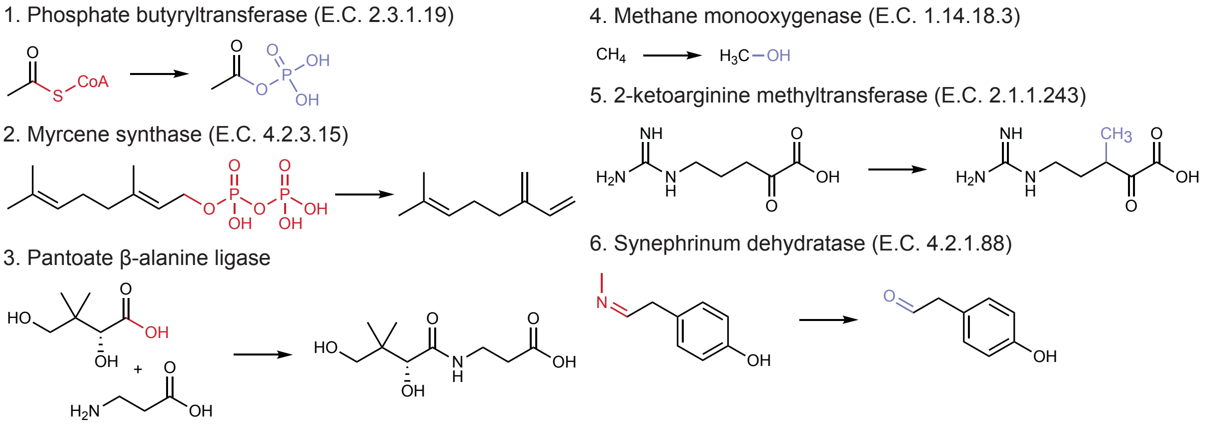

b Example of reaction templates corresponding to the transformation of a thioester to a thiol in the Reaxys template set:

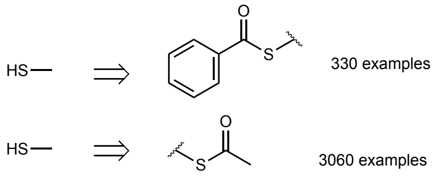

Supplementary Table 1: Precedent examples for aryl bromination reaction template

EC_Number	Enzyme Name	Reaction	SMILES	Reaction Tanimoto Similarity	Database IDs[a]
1.14.19.58	tryptophan 5-halogenase	L-tryptophan + FADH2 + Br- + O2 + H+ = 5-bromo-L-tryptophan + FAD + 2 H2O	Br.N[C@@H](Cc1c[nH]c2ccccc12)C(=O)O>>N[C@@H](Cc1c[nH]c2ccc(Br)cc12)C(=O)O	0.657	B: BS389070; K: None; M: None; S: None
1.97.1		Monobromobisphenol A <=> Bisphenol A + Hydrobromic acid	Br.CC(C)(c1ccc(O)cc1)c1ccc(O)cc1>>CC(C)(c1ccc(O)cc1)c1ccc(O)c(Br)c1	0.521	B: None; K: R06879; M: None; S: None
1.97.1		Dibromobisphenol A <=> Monobromobisphenol A + Hydrobromic acid	Br.CC(C)(c1ccc(O)cc1)c1ccc(O)c(Br)c1>>CC(C)(c1ccc(O)c(Br)c1)c1ccc(O)c(Br)c1	0.499	B: None; K: R06878; M: None; S: None
1.97.1		Tribromobisphenol A <=> Dibromobisphenol A + Hydrobromic acid	Br.CC(C)(c1ccc(O)c(Br)c1)c1ccc(O)c(Br)c1>>CC(C)(c1ccc(O)c(Br)c1)c1cc(Br)c(O)c(Br)c1	0.492	B: None; K: R06877; M: None; S: None
1.97.1		Tetrabromobisphenol A <=> Tribromobisphenol A + Hydrobromic acid	Br.CC(C)(c1ccc(O)c(Br)c1)c1cc(Br)c(O)c(Br)c1>>CC(C)(c1cc(Br)c(O)c(Br)c1)c1cc(Br)c(O)c(Br)c1	0.489	B: None; K: R06876; M: None; S: None
1.14.19.55	4-hydroxybenzoate brominase (decarboxylating)	3,4-Dihydroxybenzoate + NADPH + Bromide + Oxygen + 2 H+ <=> 3-Bromo-4,5-dihydroxybenzoate + NADP+ + 2 H2O	Br.O=C(O)c1ccc(O)c(O)c1>>O=C(O)c1cc(O)c(O)c(Br)c1	0.444	B: BR52355; K: None; M: RXN-19112; S: None
1.14.19.55	4-hydroxybenzoate brominase (decarboxylating)	4-hydroxybenzoate + NADPH + bromide + O2 + H+ = 3-bromo-4-hydroxybenzoate + NADP+ + 2 H2O	Br.O=C(O)c1ccc(O)cc1>>O=C(O)c1ccc(O)c(Br)c1	0.385	B: BR52352; K: None; M: RXN-19061; S: None
1.14.19.55	4-hydroxybenzoate brominase (decarboxylating)	2,4-dibromophenol + bromide + oxygen + NADPH + H+ = 2,4,6-tribromophenol + H2O + NADP+	Br.Oc1ccc(Br)cc1Br>>Oc1c(Br)cc(Br)cc1Br	0.357	B: None; K: None; M: RXN-19104; S: None

[a]B=Brenda, K=KEGG, M=Metacyc, S=Sabio-RK

Supplementary Figure 2: Data for the suggested enzymatic bromination of tryptoline

a Screencap of partial BRENDA entry for E.C. 1.14.19.58

SUBSTRATE	PRODUCT	REACTION DIAGRAM	ORGANISM	UNIPROT	COMMENTARY (Substrate)	LITERATURE (Substrate)
3-indolepropionate + FADH2 + chloride + O2 + H+	6-chloro-3-indolepropionate + 5-chloro-3-indolepropionate + FAD + 2 H2O		Streptomyces toxytricini	E9P162	57% conversion by the wild-type enzyme, 75% 5-chlorination is by mutant L460F/P461E/P462T in comparison to 90% 6-chlorination by the wild-type SttH	744705
anthranilamide + FADH2 + chloride + O2 + H+	5-chloro-anthranilamide + FAD + 2 H2O		Streptomyces toxytricini	E9P162	43% conversion by the wild-type enzyme	744705
anthranilate + FADH2 + chloride + O2 + H+	5-chloro-anthranilate + FAD + 2 H2O		Streptomyces toxytricini	E9P162	1.1% conversion by the wild-type enzyme	744705
indole-3-acetic acid + FADH2 + chloride + O2 + H+	5-chloroindole-3-acetic acid + FAD + 2 H2O		Streptomyces rugosporus	A4D0H5	-	744720
kynurenine + FADH2 + chloride + O2 + H+	5-chloro-kynurenine + FAD + 2 H2O		Streptomyces toxytricini	E9P162	79% conversion by the wild-type enzyme	744705
L-tryptophan + FADH2 + Br- + O2 + H+	5-bromo-L-tryptophan + FAD + 2 H2O		Streptomyces rugosporus	-	-	687866, 711742
				A4D0H5	brominating activity is about 75% of the chlorinating activity	673021
			Streptomyces rugosporus LL-42D005	A4D0H5	brominating activity is about 75% of the chlorinating activity	673021

b Amino acid sequence for PyrH (UniProt A4D0H5)

```
>tr|A4D0H5|A4D0H5_STRRG Tryptophan 5-halogenase OS=Streptomyces rugosporus
OX=295838 GN=pyrH PE=1 SV=1
MIRSVVIVGGGTAGWMTASYLKAAFDDRIDVTLVESGNVRRIGVGEATFSTVRHFFDYLG
LDEREWLPRCAGGYKLGIRFENWSEPGEYFYHPFERLRVVDGFNMAEWWLAVGDRRTSFS
EACYLTHRLCEAKRAPRMLDGSLFASQVDESLGRSTLAEQRAQFPYAYHFDADEVARYLS
EYAIARGVRHVVDDVQHVGQDERGWISGVHTKQHGEISGDLFVDCTGFRGLLINQTLGGR
FQSFSDVLPNNRAVALRVPRENDEDMRPYTTATAMSAGWMWTIPLFKRDGNGYVYSDEFI
SPEEAERELRSTVAPGRDDLEANHIQMRIGRNERTWINNCVAVGLSAAFVEPLESTGIFF
IQHAIEQLVKHFPGERWDPVLISAYNERMAHMVDGVKEFLVLHYKGAQREDTPYWKAAKT
RAMPDGLARKLELSASHLLDEQTIYPYYHGFETYSWITMNLGLGIVPERPRPALLHMDPA
PALAEFERLRREGDELIAALPSCYEYLASIQ
```

c Amino acid sequence for RebH (UniProt Q8KHZ8)

```
>sp|Q8KHZ8|REBH_LENAE Flavin-dependent tryptophan halogenase RebH OS=Lentzea
aerocolonigenes OX=68170 GN=rebH PE=1 SV=1
MSGKIDKILIVGGGTAGWMAASYLGKALQGTADITLLQAPDIPTLGVGEATIPNLQTAFF
DFLGIPEDEWMRECNASYKVAIKFINWRTAGEGTSEARELDGGPDHFYHSFGLLKYHEQI
PLSHYWFDRSYRGKTVEPFDYACYKEPVILDANRSPRRLDGSKVTNYAWHFDAHLVADFL
RRFATEKLGVRHVEDRVEHVQRDANGNIESVRTATGRVFDADLFVDCSGFRGLLINKAME
EPFLDMSDHLLNDSAVATQVPHDDDANGVEPFTSAIAMKSGWTWKIPMLGRFGTGYVYSS
RFATEDEAVREFCEMWHLDPETQPLNRIRFRVGRNRRAWVGNCVSIGTSSCFVEPLESTG
IYFVYAALYQLVKHFPDKSLNPVLTARFNREIETMFDDTRDFIQAHFYFSPRTDTPFWRA
NKELRLADGMQEKIDMYRAGMAINAPASDDAQLYYGNFEEEFRNFWNNSNYYCVLAGLGL
VPDAPSPRLAHMPQATESVDEVFGAVKDRQRNLLETLPSLHEFLRQQHGR
```

Supplementary Table 2: Parameters used for full synthesis plan searches

expansion_time	180
filter_threshold	0.0
max_cum_prob	0.9999
return_first	false
max_depth	10
template_count	1000
max_branching	25
max_ppg	100
return_graph	true

Supplementary Table 3: Automatically determined cofactor pairs

If a molecule from the column Cofactor 1 and at least one of the molecules from the column Cofactor 2 were present on opposite sides of a reaction, both were removed before converting the reaction into SMILES strings. All names were converted to lower case to increase consistency.

**Cofactor 1**	**Cofactor 2**
(2e,6e)-farnesyl diphosphate	diphosphate
gdp-beta-l-fucose	gdp
ferricytochrome c	ferrocytochrome c
malonyl-[acp]	soluble [acyl-carrier protein], co2
reduced adrenodoxin	h2o, oxidized adrenodoxin
acceptor	reduced acceptor
gdp-alpha-d-mannose	gdp
udp-alpha-d-glucose	udp
fmnh2	fmn
dimethylallyl diphosphate	diphosphate
protein n(pi)-phospho-l-histidine	protein histidine
oxidized ferredoxin [iron-sulfur] cluster	h+, reduced ferredoxin [iron-sulfur] cluster
udp-d-glucuronate	udp
reduced [nadph-hemoprotein reductase]	h2o, oxidized [nadph-hemoprotein reductase]
oxidized ferredoxin	reduced ferredoxin
udp-galactose	udp
udp-glucuronate	udp
udp-glucose	udp
electron-transfer quinone	electron-transfer quinol
ferrocytochrome b5	ferricytochrome b5, h2o
udp-n-acetyl-d-glucosamine	udp
[hpr protein]-npi-phospho-l-histidine	[hpr]-l-histidine
nadph	nadp+
3'-phosphoadenylylsulfate	adenosine 3',5'-bisphosphate
3'-phosphoadenylyl sulfate	adenosine 3',5'-bisphosphate
[protein]-npi-phospho-l-histidine	[protein]-l-histidine
prenyl diphosphate	diphosphate
(s)-methylmalonyl-coa	coenzyme a, nadp+, co2
dolichyl phosphate d-mannose	dolichyl phosphate
oxidized unknown electron carrier	reduced unknown electron carrier
isopentenyl diphosphate	diphosphate
electron-transfer flavoprotein	reduced electron-transfer flavoprotein
reduced acceptor	acceptor

fadh2	fad
udp-d-galactose	udp
[reduced nadph-hemoprotein reductase]	[oxidized nadph-hemoprotein reductase], h2o
reduced unknown electron carrier	oxidized unknown electron carrier
nad+	nadh
udp-alpha-d-galactose	udp
nadh	nad+
udp-alpha-d-xylose	udp
nadp+	nadph
oxidized c-type cytochrome	h+, reduced c-type cytochrome
hydrogen peroxide	oxygen, h2o
3'-phosphoadenylyl-sulfate	adenosine 3,5'-bisphosphate
fad	fadh2
udp-beta-l-rhamnose	udp
ah2	h2o, a
udp-n-acetyl-d-galactosamine	udp
reduced ferredoxin [iron-sulfur] cluster	oxidized ferredoxin [iron-sulfur] cluster
reduced ferredoxin	oxidized ferredoxin
cmp-n-acetyl-beta-neuraminate	cmp
cmp-n-acetylneuraminate	cmp
udp-alpha-d-glucuronate	udp
oxidized electron-transfer flavoprotein	reduced electron-transfer flavoprotein
udp-d-xylose	udp
gdp-l-fucose	gdp
na+	na+
[reduced nadph---hemoprotein reductase]	[oxidized nadph---hemoprotein reductase], h2o
ubiquinone	ubiquinol
[hpr]-l-histidine	[hpr protein]-npi-phospho-l-histidine
[protein]-l-histidine	[protein]-npi-phospho-l-histidine
[oxidized nadph---hemoprotein reductase]	oxygen, [reduced nadph---hemoprotein reductase]
glutathione disulfide	glutathione
protein histidine	protein n(pi)-phospho-l-histidine
nicotinamide	nad+
ferrocytochrome c	ferricytochrome c
ubiquinol	ubiquinone
electron-transfer quinol	electron-transfer quinone
quinol	quinone
h2o2	o2
oxidized [nadph-hemoprotein reductase]	oxygen, reduced [nadph-hemoprotein reductase]

reduced c-type cytochrome	oxidized c-type cytochrome
a	ah2
oxidized flavodoxin	reduced flavodoxin
s-adenosyl-l-homocysteine	s-adenosyl-l-methionine
5'-deoxyadenosine	s-adenosyl-l-methionine
2'-o-acetyl-adp-ribose	nad+
adp	atp
ferricytochrome b5	h+, ferrocytochrome b5
[oxidized nadph-hemoprotein reductase]	[reduced nadph-hemoprotein reductase], o2

Additionally, all of the following entities were removed:
h+, e-, atp, hnu, h2o, water, oxygen, o2, adp, amp, phosphate, diphosphate, co2, sodium, na+, ammonium, nh3

Supplementary Figure 3: Molecules for which all routes found required a transformation that could not be described by the synthetic chemistry template set

Molecules from the ZINC boutique subset. Routes were identified for these molecules when the retrosynthetic search was performed using both the enzymatic and synthetic template prioritizers but not when the search was performed with only the synthetic template prioritizer. Additionally, all of the routes found for these molecules required at least one reaction that could not be described by the set of synthetic reaction rules from Reaxys.

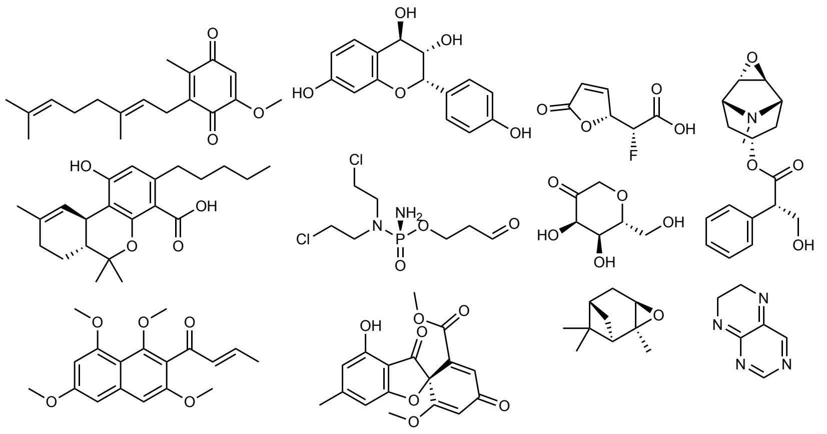

Supplementary Figure 4: Molecules for which routes were found with a hybrid search but not with a fully synthetic or fully enzymatic search

Molecules from the ZINC boutique subset. Routes were identified for these molecules when the retrosynthetic search was performed using both the enzymatic and synthetic template prioritizers but not when the search was performed with only one template prioritizer at once.

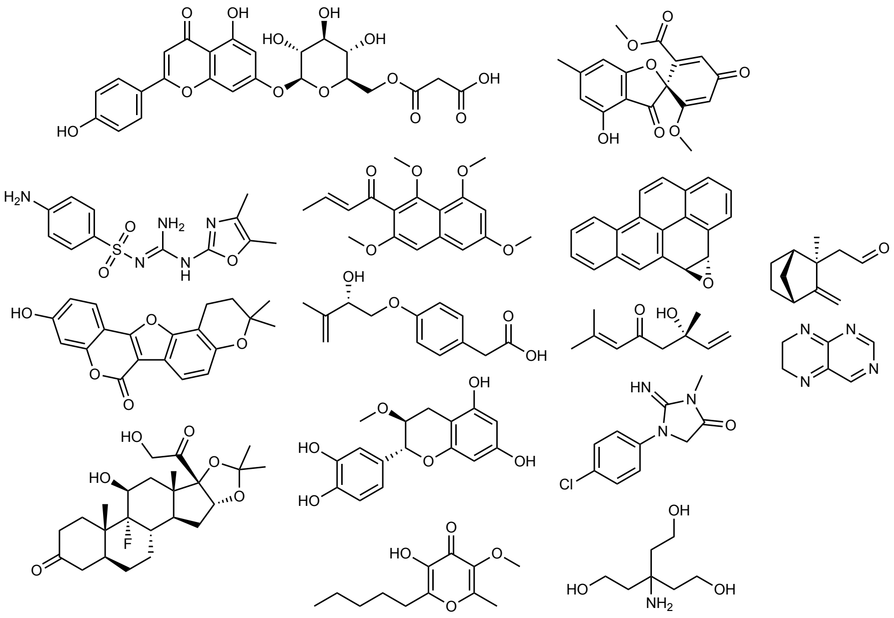

Supplementary Figure 5: Screenshot of synthesis route identified for dronabinol ((-)-1)

The synthesis route is visualized in the ASKCOS graphical user interface (GUI). One synthesis tree is displayed at a time. Molecule nodes are framed in red if they are not in the buyable database, and yellow if they are in the buyable database. In this image, the final reaction node is selected (highlighted in blue in the left pane), and the corresponding information is shown in the pane on the right. "bkms" under "Supporting templates" indicates that the precedent for this reaction is from the BKMS database. The template ID written in blue text links to additional information about the template.

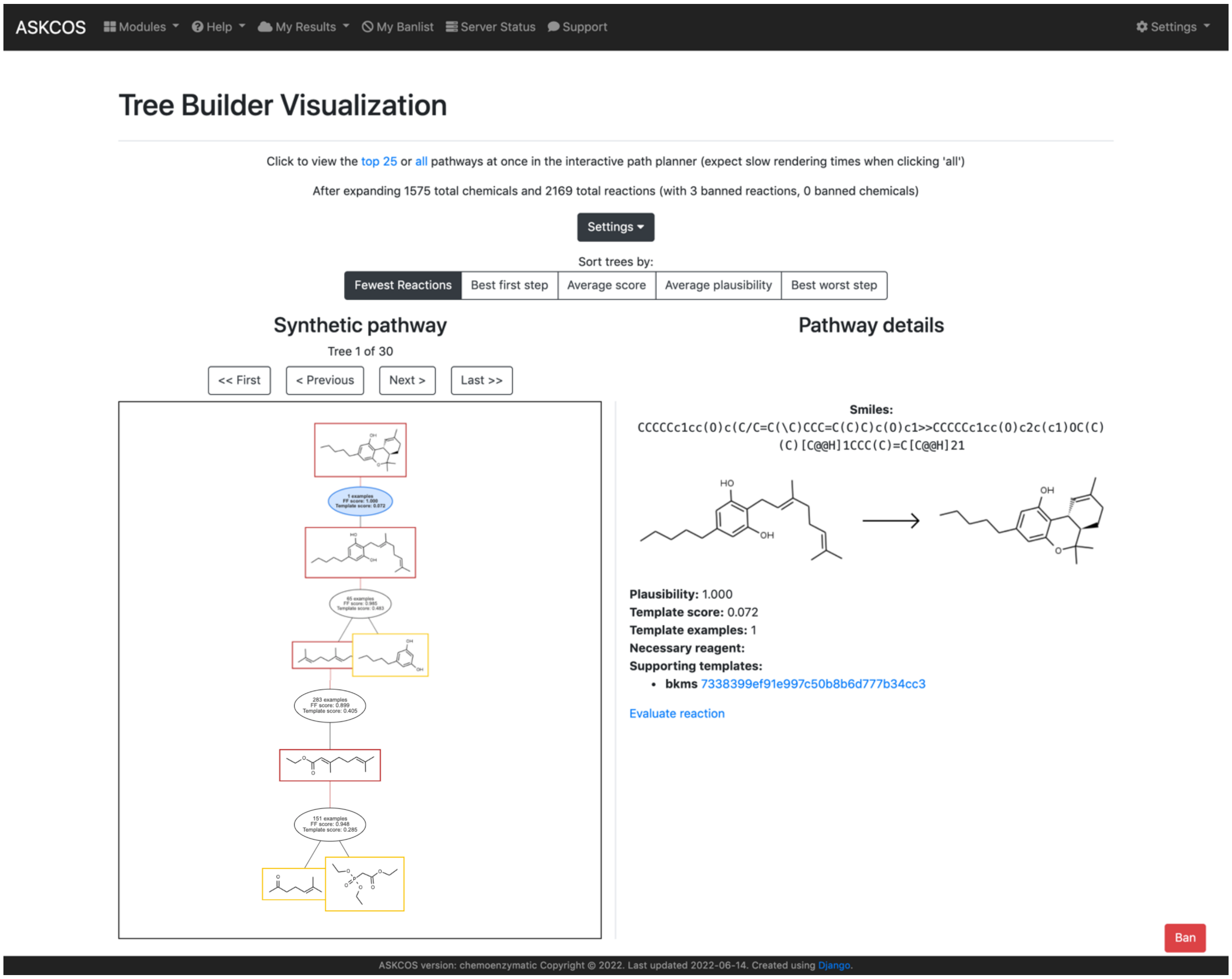

Supplementary Figure 6: Screenshots for the 6 shortest hybrid synthesis routes identified for arformoterol ((R,R)-2)

The synthesis routes are visualized with the ASKCOS graphical user interface. While the routes vary in length and starting materials, they follow a common synthesis logic described in the main text. Molecules were redrawn in ChemDraw and pasted on the screenshots for higher image resolution.

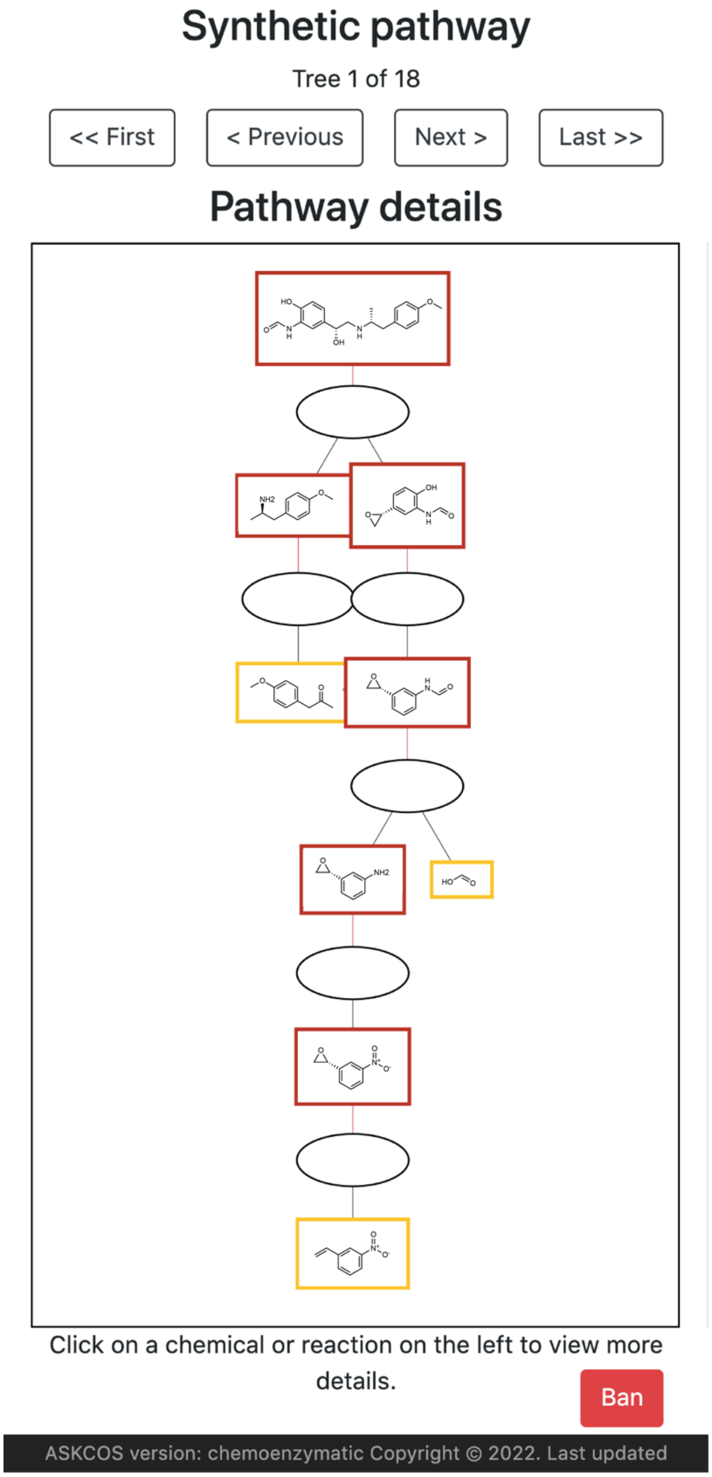

Synthetic pathway

Tree 2 of 18

<< First   < Previous   Next >   Last >>

Pathway details

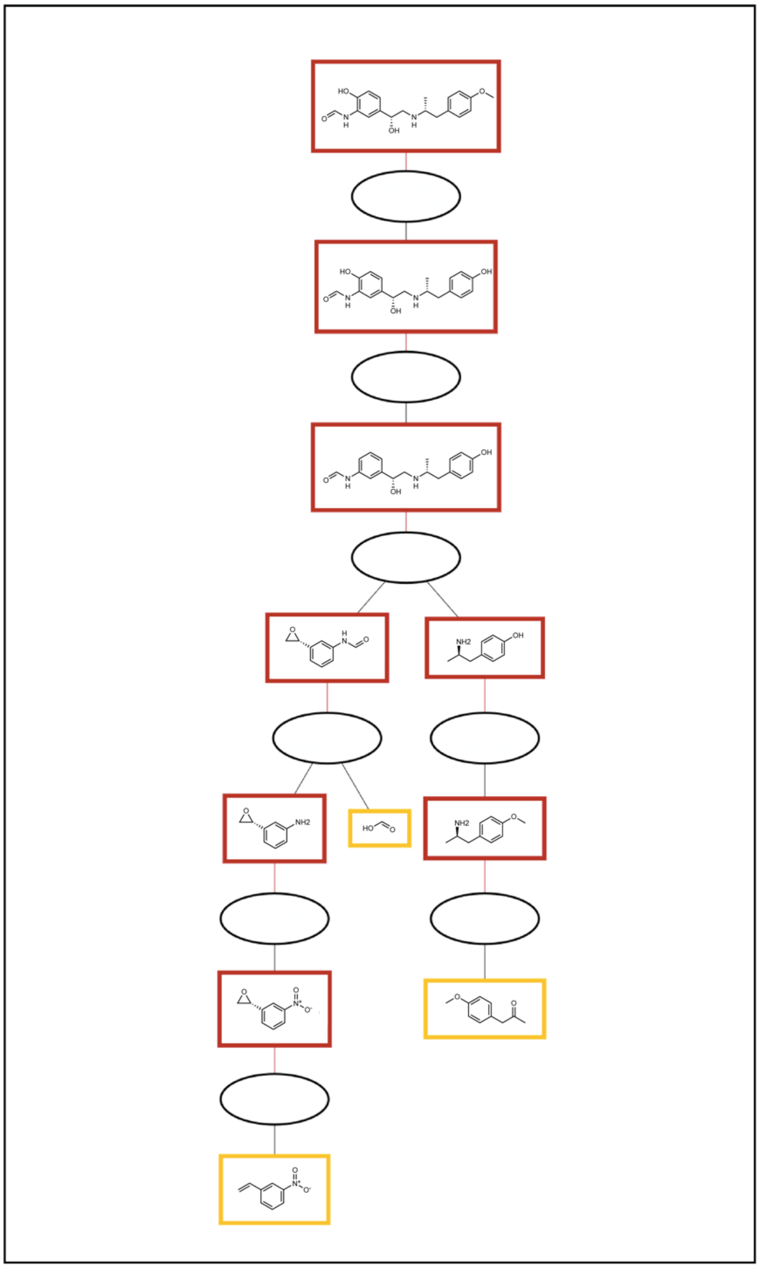

Click on a chemical or reaction on the left to view more details.

Ban

ASKCOS version: chemoenzymatic Copyright © 2022. Last updated

Synthetic pathway

Tree 3 of 18

<< First   < Previous   Next >   Last >>

Pathway details

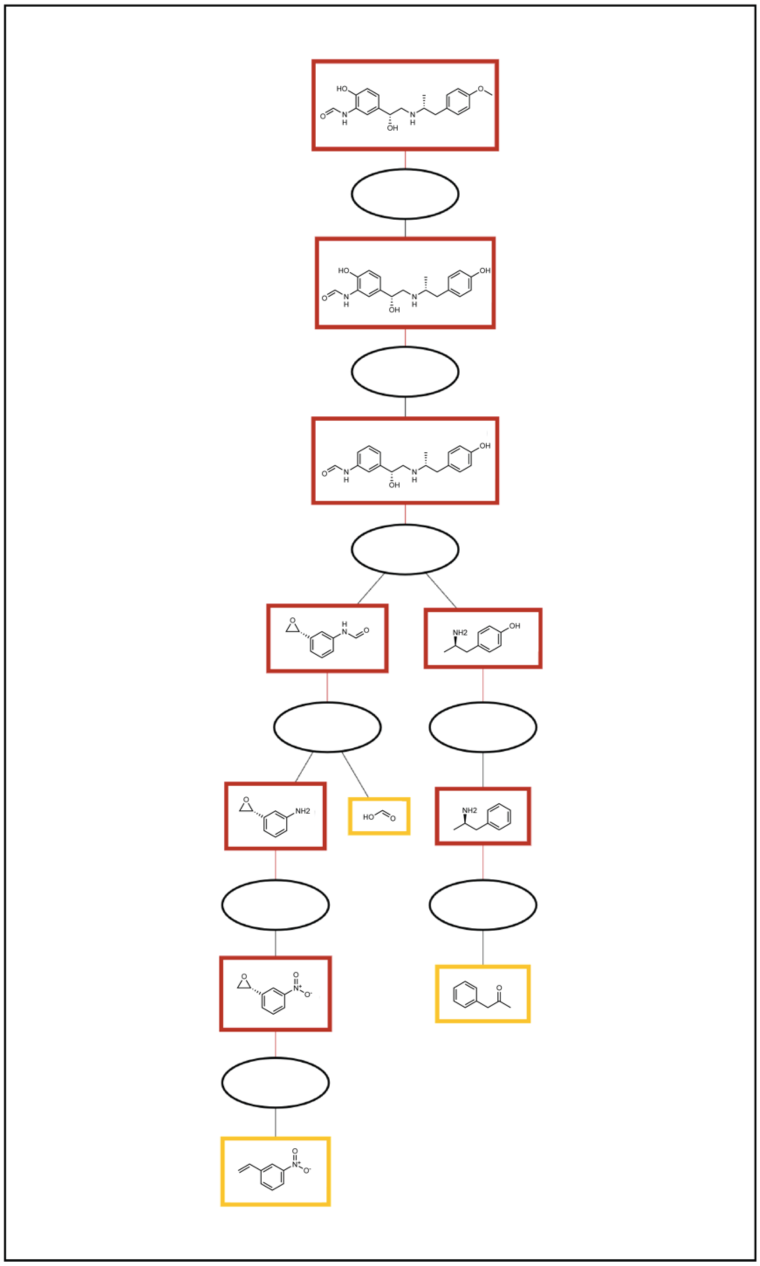

Click on a chemical or reaction on the left to view more details.

Ban

ASKCOS version: chemoenzymatic Copyright © 2022. Last updated

Synthetic pathway

Tree 4 of 18

<< First < Previous Next > Last >>

Pathway details

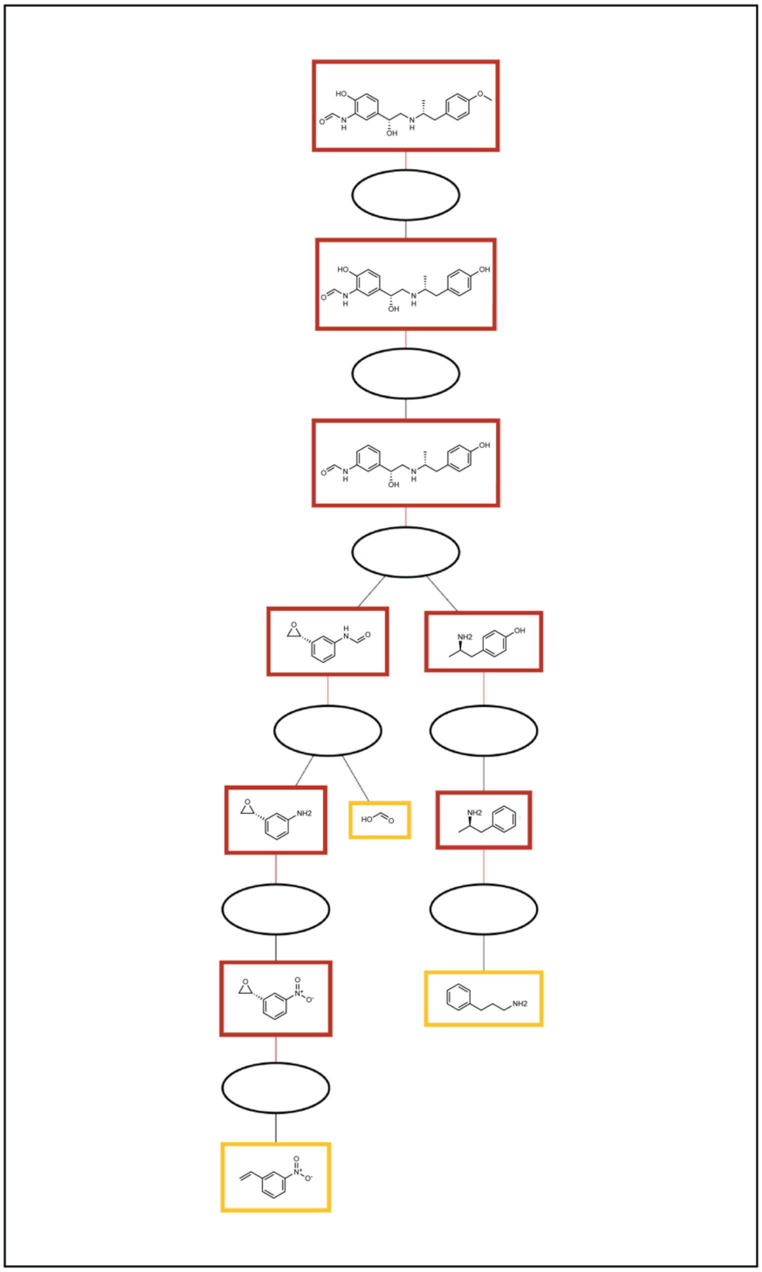

Click on a chemical or reaction on the left to view more details.

Ban

ASKCOS version: chemoenzymatic Copyright © 2022. Last updated

Synthetic pathway

Tree 5 of 18

<< First | < Previous | Next > | Last >>

Pathway details

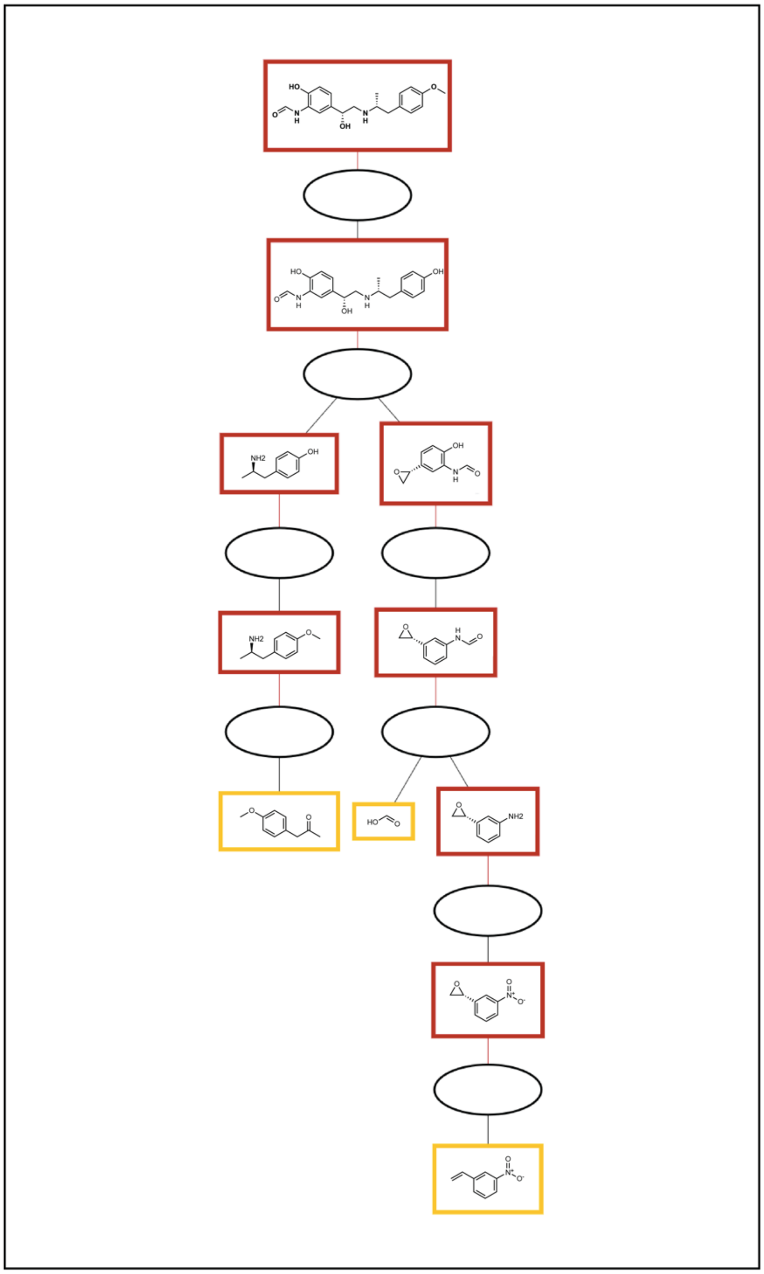

Click on a chemical or reaction on the left to view more details.

Ban

ASKCOS version: chemoenzymatic Copyright © 2022. Last updated

Synthetic pathway

Tree 6 of 18

<< First | < Previous | Next > | Last >>

Pathway details

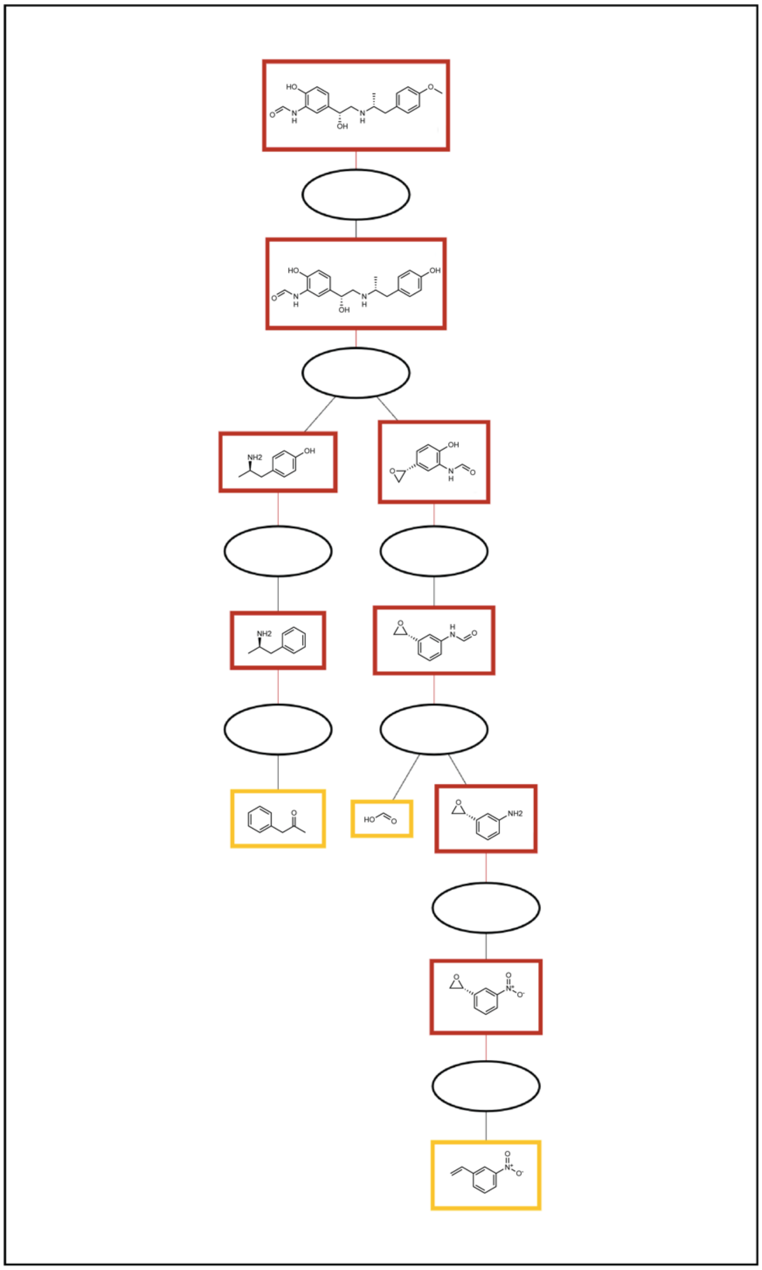

Click on a chemical or reaction on the left to view more details.

Ban

ASKCOS version: chemoenzymatic Copyright © 2022. Last updated

Supplementary Figure 7: Retrosynthesis prediction accuracy on test set as a function of number of training examples seen for a template

The reaction data are the 15,309 reactions from BKMS labeled with 7,984 extracted templates. The evaluation was performed with a neural network model trained on 80% of the reactions and tested on 10% of the reactions where the data was split into train, validation, and test splits using a stratified split. The accuracies are reported as the fraction of molecules whose template is seen a given number of times during training where the template is ranked among the top-k templates by the template prioritizer model. The pretrained model was first trained to predict template applicability for each product molecule whereas the baseline model was not. The dashed lines represent the overall accuracies of each model.

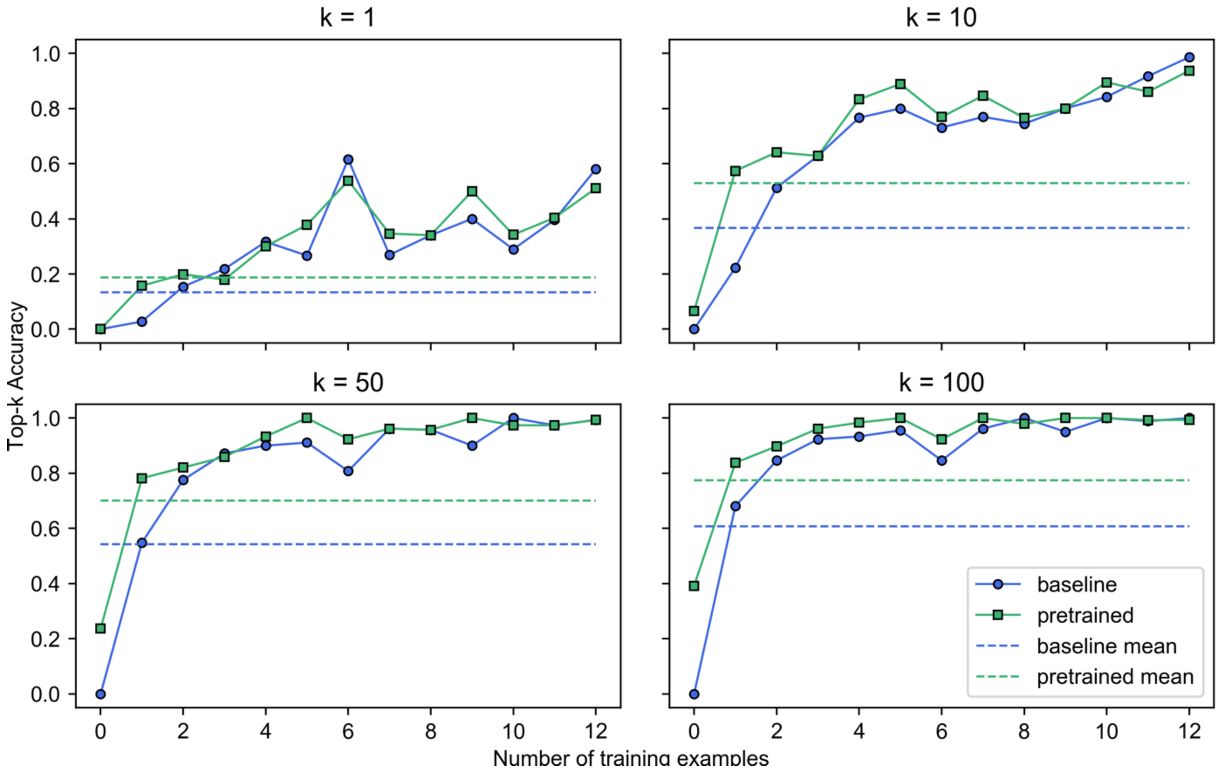

Supplementary Figure 8: Accuracy metrics during training of the enzymatic one-step model

Metrics during training of the single-step enzymatic retrosynthesis network trained on 80% of the BKMS reaction data and validated on 10% of the data, pretrained on template applicability.

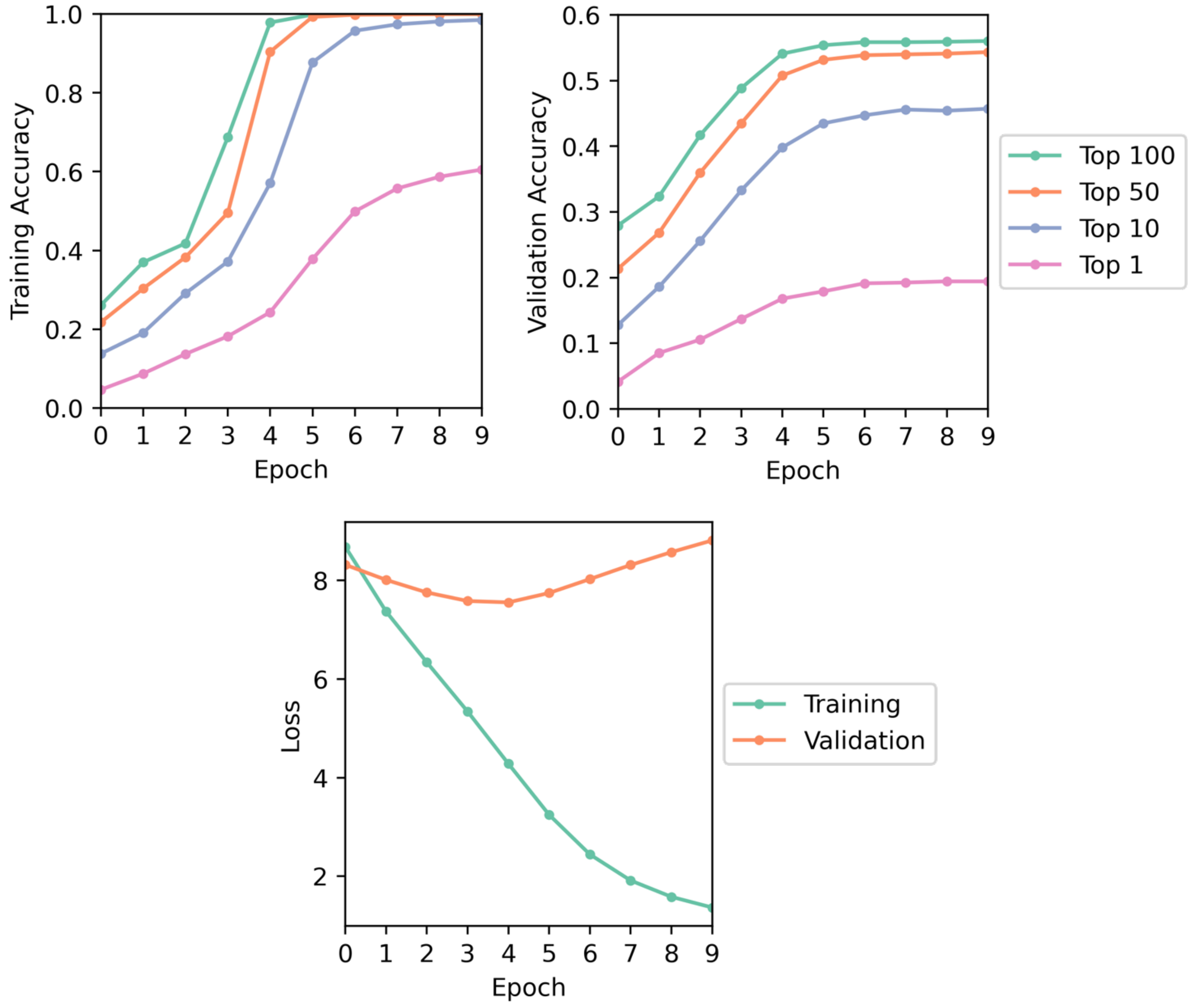

Supplementary Figure 9: Number of nodes explored for multi-step retrosynthetic search with different search strategies

Mean number of chemical (**a**) and reaction (**b**) nodes explored in a 3-minute time-limited search with the parameters described in Supplementary Table 2 for 1,000 compounds from the ZINC boutique dataset. Error bars represent ±1 standard deviation. We hypothesize that the enzymatic search explores more nodes both because the template prioritizer model is much smaller than the synthetic template prioritizer model, so more inference calls can be made within a given time limit, and it is possible that the enzymatic templates are less redundant, so less time is wasted re-exploring chemical nodes that have already been explored during the search.

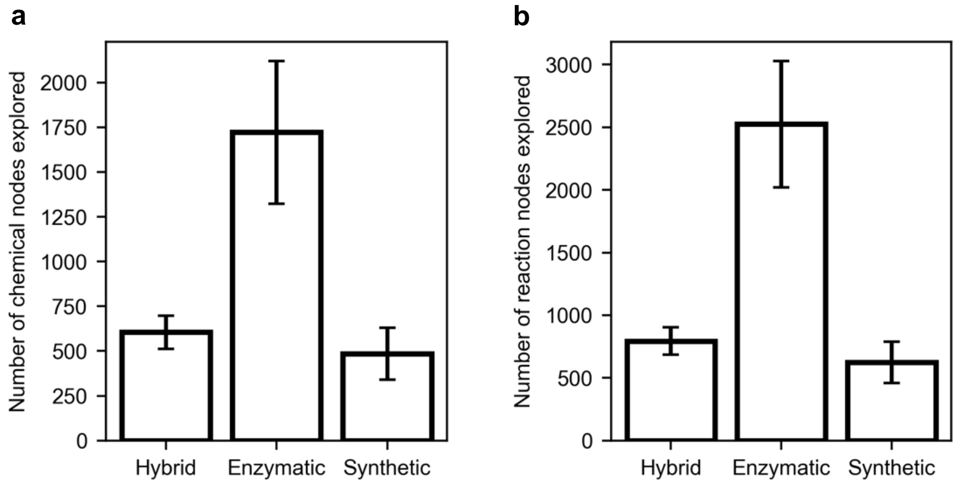

Supplementary Figure 10: Comparing interactive retrosynthetic search results to experimental pathways for islatravir and sitagliptin

The rank of the individual steps from experimentally validated pathways is shown when using the synthetic (S), enzymatic (E), and both (hybrid, H) template prioritizer models to suggest precursors. “NA” indicates that none of the top-1000 ranked templates reproduce the reaction. The reported rank is the rank of the template which recovers the experimental step when templates that cannot be applied to the product are removed from the suggestions.
The top three suggested precursors when suggestions from the enzymatic and synthetic models are combined are shown below the experimental pathways. The substructure of the precursor that is different from the product is highlighted in red. If a template generates more than one precursor, only one precursor is depicted.

a Sitagliptin reaction demonstrated by Savile et al.[1]

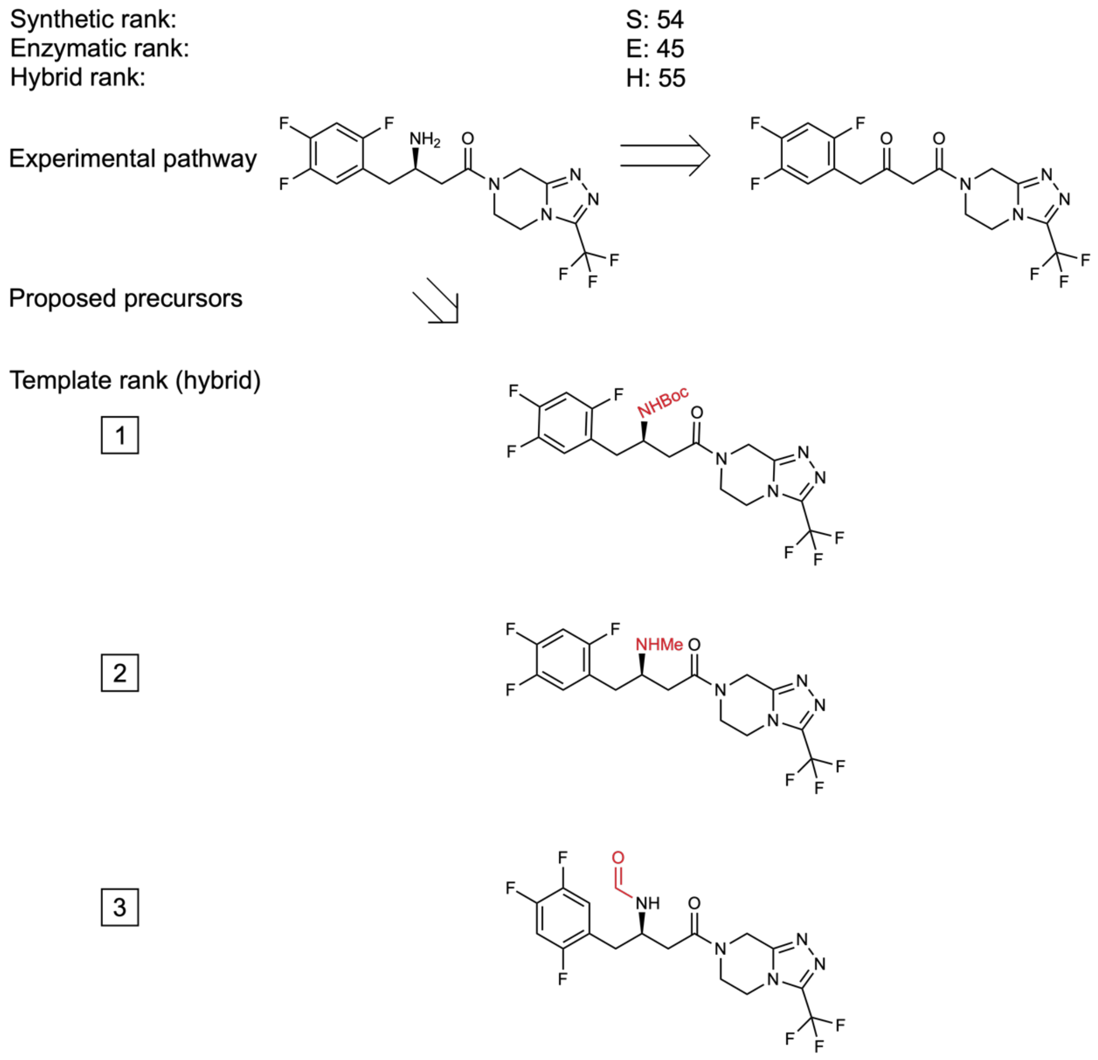

b Islatravir pathway demonstrated by Huffman et al.[2]
Stereochemistry was included at the C1 position for the precursor of the first retrosynthetic step when ranking the step for the synthetic model but not the enzymatic model to match the stereochemistry defined in the top-ranked templates from the respective template sets for the reaction. This discrepancy is minor and reasonable to catch in the interactive search context.

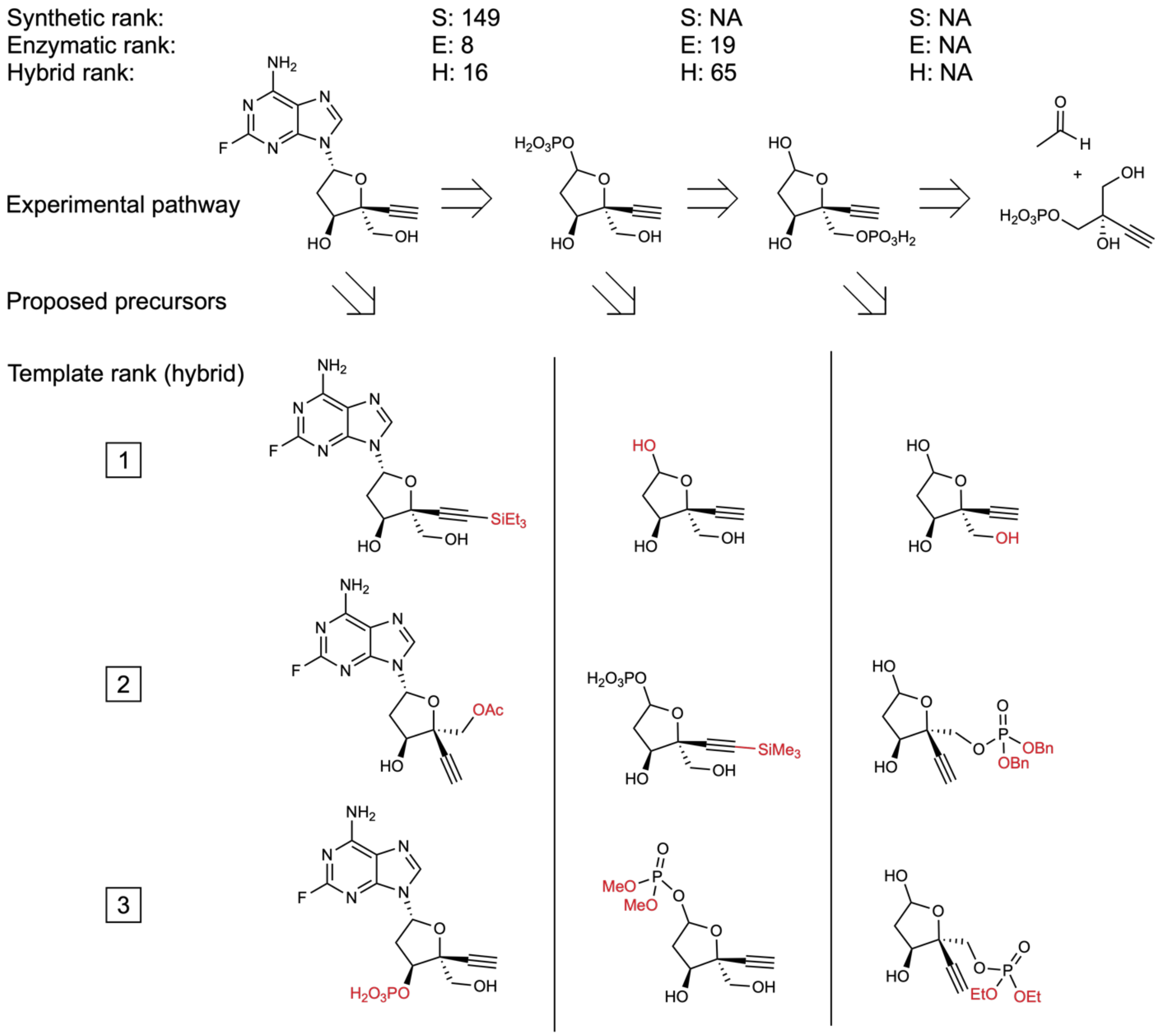

References

(1) Savile, C. K.; Janey, J. M.; Mundorff, E. C.; Moore, J. C.; Tam, S.; Jarvis, W. R.; Colbeck, J. C.; Krebber, A.; Fleitz, F. J.; Brands, J.; Devine, P. N.; Huisman, G. W.; Hughes, G. J. Biocatalytic Asymmetric Synthesis of Chiral Amines from Ketones Applied to Sitagliptin Manufacture. *Science* **2010**, *329* (5989), 305–309. https://doi.org/10.1126/science.1188934.

(2) Huffman, M. A.; Fryszkowska, A.; Alvizo, O.; Borra-Garske, M.; Campos, K. R.; Canada, K. A.; Devine, P. N.; Duan, D.; Forstater, J. H.; Grosser, S. T.; Halsey, H. M.; Hughes, G. J.; Jo, J.; Joyce, L. A.; Kolev, J. N.; Liang, J.; Maloney, K. M.; Mann, B. F.; Marshall, N. M.; McLaughlin, M.; Moore, J. C.; Murphy, G. S.; Nawrat, C. C.; Nazor, J.; Novick, S.; Patel, N. R.; Rodriguez-Granillo, A.; Robaire, S. A.; Sherer, E. C.; Truppo, M. D.; Whittaker, A. M.; Verma, D.; Xiao, L.; Xu, Y.; Yang, H. Design of an in Vitro Biocatalytic Cascade for the Manufacture of Islatravir. *Science* **2019**, *366* (6470), 1255–1259. https://doi.org/10.1126/science.aay8484.
